# Integrated gut microbiome and metabolomic analyses elucidate the therapeutic mechanisms of Suanzaoren decoction in insomnia and depression models

**DOI:** 10.3389/fnins.2024.1459141

**Published:** 2024-10-11

**Authors:** Hongxiong Zhang, Taixiang Gao, Feng Zhao, Nan Wang, Zhixuan Li, Xuemei Qin, Ying Liu, Rui Wang

**Affiliations:** ^1^College of Traditional Chinese Medicine and Food Engineering, Shanxi University of Chinese Medicine, Jinzhong, China; ^2^Wuxi School of Medicine, Jiangnan University, Wuxi, China; ^3^Modern Research Center for Traditional Chinese Medicine, Shanxi University, Taiyuan, China; ^4^Beijing Research Institute of Chinese Medicine, Beijing University of Chinese Medicine, Beijing, China

**Keywords:** Suanzaoren decoction, insomnia, depression, gut microbiome, metabolomics

## Abstract

Insomnia and depression are psychiatric disorders linked to substantial health burdens. The gut microbiome and metabolomic pathways are increasingly recognized as key contributors to these conditions’ pathophysiology. Suanzaoren Decoction (SZRD), a traditional Chinese herbal formulation, has demonstrated significant therapeutic benefits for both insomnia and depression. This study aims to elucidate the mechanistic effects of SZRD on insomnia and depression by integrating gut microbiome and metabolomic analyses and to assess the differential impacts of SZRD dosages. Using ultra-high-performance liquid chromatography-mass spectrometry (UHPLC–MS), we identified 66 chemical constituents within SZRD. Behavioral assays indicated that low-dose SZRD (LSZRD) significantly ameliorated insomnia symptoms in rat models, whereas high-dose SZRD (HSZRD) markedly improved depressive behaviors. 16S rRNA sequencing revealed that SZRD modulated gut microbiome dysbiosis induced by insomnia and depression, characterized by an increased abundance of short-chain fatty acid (SCFA)-producing genera. Metabolomic profiling demonstrated reduced plasma amino acid metabolites and disrupted *γ*-aminobutyric acid (GABA) and L-glutamic acid metabolism in the hippocampus of affected rats. SZRD administration restored fecal SCFA levels and ameliorated metabolic imbalances in both plasma and hippocampal tissues. These findings underscore the pivotal role of gut microbiome modulation and metabolic regulation in the therapeutic effects of SZRD, providing a scientific basis for its use in treating insomnia and depression.

## 1 Introduction

Insomnia, the most prevalent sleep disorder, severely disrupts both neurological and physiological functions due to insufficient sleep. It is linked to an increased risk of various health conditions, including cardiovascular disease, hypertension, chronic heart failure, and depression (Perlis et al., 2022). Cognitive-Behavioral Therapy for Insomnia (CBT-I) and benzodiazepines are the primary treatments for insomnia. However, long-term benzodiazepine use can lead to tolerance and cognitive decline (Shi et al., 2022). Depression, an increasingly prevalent mental illness worldwide, manifests as persistent low mood, anhedonia, cognitive impairments, and attention deficits (McCarron et al., 2021). Selective Serotonin Reuptake Inhibitors (SSRIs) are the cornerstone of pharmacological treatment for depression but carry potential side effects and dependency risks (Ślifirski et al., 2021).

Traditional Chinese Medicine (TCM) offers unique therapeutic benefits for psychiatric conditions such as insomnia and depression (Bi et al., 2022). Suanzaoren Decoction (SZRD), originating from Zhang Zhongjing’s Synopsis of the Golden Chamber, comprises processed Ziziphi Spinosae Semen (Chaosuanzaoren), Poria (Fuling), Anemarrhenae Rhizoma (Zhimu), Chuanxiong Rhizoma (Chuanxiong), and Glycyrrhizae Radix et Rhizoma (Gancao). SZRD has demonstrated sedative-hypnotic properties and is effective in improving sleep disturbances and regulating the contents of hypothalamic neurotransmitters 5-HT, NE, and DA in insomnia model (Dong et al., 2021). Additionally, it significantly alleviates anxiety-like symptoms and improves 5-HT, GABA and NE levels in the hippocampus of mice subjected to chronic restraint stress (Yan et al., 2023). Controlled clinical trials have shown that SZRD alone as well as in combination with western medicine has a higher clinical effective rate in the treatment of insomnia than western medicine alone (e.g., the benzodiazepines) (Xie et al., 2013). It has been pointed out that SZRD can exert antidepressant effects by reducing levels of inflammatory factors and regulating neuroplasticity (Du et al., 2024). In clinical practice, many TCM have antidepressant effects (Ma et al., 2020), the licorice total flavonoids (LF) and liquiritin in SZRD are the material basis for their antidepressant effects (Wang et al., 2023). Our prior research indicates that SZRD affects the levels of Noradrenaline (NE), Dopamine (DA), and 5-hydroxytryptamine (5-HT) in insomnia and depression models, with low-dose SZRD (LSZRD) being more effective for insomnia and high-dose SZRD (HSZRD) for depression (Zhang et al., 2023).

Emerging evidence underscores the gut microbiome’s pivotal role in central nervous system disorders (Wang Z. et al., 2022). Key communication pathways, including the vagus nerve, tryptophan metabolites, and microbial products like short-chain fatty acids (SCFAs) and peptidoglycans, mediate gut-brain interactions, influencing serotonergic, noradrenergic, dopaminergic, glutamatergic, and GABAergic neurotransmission (Socała et al., 2021). The gut microbiome modulates behaviors such as anxiety, depression, and cognitive function (Foster and McVey Neufeld, 2013). Metabolic abnormalities in insomnia have been linked to disruptions in gut microbiome-associated serum metabolites (Wang Q. et al., 2022). Probiotic supplementation has shown promise in restoring gut metabolic balance and ameliorating symptoms of depression and anxiety (Zhu et al., 2023).

Although the connection between gut microbiome alterations and central nervous system function is well-established (Ma et al., 2019), little is known about differences in gut microbiome changes triggered by the two psychiatric disorders, insomnia and depression. In addition, metabolic differences resulting from insomnia and depression warrant further investigation, and based on the same therapeutic agent, SZRD, it is necessary to understand the specific changes of SZRD on the gut microbiome and metabolome in two models of insomnia and depression. Therefore, we propose the scientific hypothesis that different doses of SZRD modulate the gut microbiome and metabolome to improve insomnia and depressive symptoms. This study aims to elucidate the therapeutic effects of SZRD on insomnia and depression models, focusing on the modulation of the gut microbiome and metabolomic profiles. We employed separate rat models of insomnia and depression to evaluate symptom improvement with varying SZRD doses. Gut microbiome composition was analyzed using 16S rRNA sequencing, and the effects of SZRD on plasma and hippocampal metabolites were assessed via gas chromatography–mass spectrometry (GC–MS). Our findings suggest that different doses of SZRD modulates the gut microbiome and metabolic pathways in insomnia and depression models, supporting low-dose SZRD for insomnia and high-dose SZRD for depression treatment.

## 2 Materials and methods

### 2.1 Herbal medicines

SZRD comprises the following botanical ingredients: processed *Ziziphus jujuba* Mill. var. *spinosa* (Bunge) Hu ex H. F. Chou (Chaosuanzaoren), *Poria cocos* (Schw.) Wolf (Fuling), *Anemarrhena* *asphodeloides* Bge. (Zhimu), *Ligusticum chuanxiong* Hort. (Chuanxiong), and *Glycyrrhiza uralensis* Fisch. (Gancao). These herbs were purchased from Shanxi Yuanhetang Traditional Chinese Medicine Co., Ltd., under batch numbers 230,102, 210,901, 210,901, 210,802, and 200,401, respectively. The authenticity of each herb was confirmed by Professor Xiangping Pei from Shanxi University of Traditional Chinese Medicine, following the identification protocols specified in the Chinese Pharmacopoeia (2020 Edition). Voucher specimens (No. SXTCM-Wang-2022001 for Chaosuanzaoren, No. SXTCM-Wang-2022002 for Fuling, No. SXTCM-Wang-2022003 for Chuanxiong, No. SXTCM-Wang-2022004 for Zhimu. and No. SXTCM-Wang-2022005 for Gancao) have been deposited in the public Herbarium of Shanxi University of Traditional Chinese Medicine.

For the preparation of SZRD, the herbs were mixed in a specific ratio of 15:6:6:6:6:3. This mixture was immersed in water, using nine times the volume of the herbal mixture, and allowed to soak for 30 min to ensure thorough hydration. Subsequently, the mixture was subjected to three rounds of reflux extraction, each lasting 1.5 h, to maximize the extraction of active compounds. The resultant extract was filtered through gauze to remove particulate matter and concentrated at 60°C until the desired consistency of 0.36 g/mL to 3.60 g/mL was achieved.

### 2.2 UHPLC-LTQ-Orbitrap-MS analysis

The SZRD solution (0.218 g/mL) was prepared according to the procedure described in section 2.1. The solution was subsequently centrifuged at 14,000 rpm for 5 min, and the supernatant was filtered through a 0.22-μm microporous filter membrane. For the UHPLC–MS analysis, an Ultimate 3,000 UHPLC system coupled with an LTQ-Orbitrap XL Mass Spectrometer (Thermo Fisher Scientific, United State) was utilized. Chromatographic separation was achieved using an ACQUITY UPLC BEH C18 column (100 mm × 2.1 mm, 1.7 μm). The mobile phases consisted of 0.1% formic acid in ultrapure water (solvent A) and acetonitrile (solvent B). The gradient elution program initiated with 95% solvent A, gradually decreasing to 83% over the first 8 min. It then maintained at 83% for 2 min, followed by a gradual reduction to 82% in the next minute, and further decreased to 80% within the following minute. The solvent A percentage continued to decline to 77% over the next 5 min, then to 67% over the subsequent 5 min. From 22 to 30 min, the percentage of solvent A decreased to 40%, followed by a rapid decline to 0% over the next 2 min. The column was then maintained at 0% solvent A for 4 min before being re-equilibrated back to 95% over the last 6 min. The flow rate was consistently maintained at 0.3 mL/min, with the column temperature set at 30°C and an injection volume of 3 μL.

Mass spectrometric detection employed an electrospray ionization (ESI) source in both positive and negative ion modes. Nitrogen, with a purity>99.99%, was used as the sheath gas at a flow rate of 40 arb and as the auxiliary gas at a flow rate of 20 arb. Helium, also with a purity>99.99%, served as the collision gas. The ionization source voltage was maintained at 3.0 kV, the ion source temperature at 350°C, the tube lens voltage at 110 V, and the drying gas flow rate at 15 L/min. Collision voltages ranged from 6 to 10 V. Primary mass spectra were obtained using Fourier Transform high-resolution full scan (FT) mode, covering a mass range of m/z 100 to 1,200 with a resolution of 30,000. Secondary mass spectra were acquired through data-dependent scanning (DDS), selecting the three most abundant ions from the primary scan for collision-induced dissociation (CID) fragmentation. The CID activation Q was set at 0.25, with an activation time of 30 ms and a normalized collision energy of 35%. The raw data were processed using Xcalibur 4.1 software. The primary mass spectra of chromatographic peaks were compared against the ChemSpider database to predict molecular weights, molecular formulas, and structural formulas. The secondary or multistage mass spectra were analyzed using the MassBank database to identify compounds within a 5 ppm error range, corroborated by literature reports.

### 2.3 Animals

In this study, specific pathogen-free (SPF) male Sprague–Dawley (SD) rats, with a body weight of 200 ± 20 g, were obtained from SPF (Beijing) Biotechnology Co., Ltd. [Production license number: SCXK (Jing) 2019–0010]. The rats were housed in the animal facility at Shanxi University of Traditional Chinese Medicine, where environmental conditions were rigorously controlled. The temperature in the facility was maintained between 23 and 26°C. The rats had unrestricted access to standard laboratory chow and water.

### 2.4 Preparation and dosing regimen of insomnia and depression rat models

Following a 7-day acclimatization period, 50 SD rats were randomly assigned to five groups (*n* = 10 per group): Control, Insomnia, LSZRD (3.6 g/kg/day), HSZRD (36 g/kg/day), and Diazepam (0.5 mg/kg/day; batch number 20220501, China). Except for the control group, all groups received intraperitoneal injections of para-chlorophenylalanine (PCPA; Aladdin, batch number C2208166, China) suspension for two consecutive days between 9:00 and 10:00 a.m. The control group received weak alkaline saline injections. PCPA was dosed at 400 mg/kg, prepared by finely grinding the powder, mixing it with saline, adding an appropriate amount of carboxymethyl cellulose sodium (CMC-Na; Kermel, batch number 20150110, China), and adjusting the pH to 7–8 with stirring.

For the depression model, another set of 50 SD rats was randomly divided into five groups: Control, Depression, LSZRD (3.6 g/kg/day), HSZRD (36 g/kg/day), and Xiaoyaopill (0.9 g/kg/day; batch number 1906066, China). Except for the control group, the rats were individually housed and exposed to one of the following daily stressors: 12 h of food or water deprivation, 24 h of cohabitation with another rat, 5 min at 45°C, tail clamping with hemostatic forceps for 1 min, 12 h on wet bedding, 6 min of cold swimming at 10°C, 12 h in a 45° tilted cage, 12 h with foreign object interference, or 3 h of whole-body restraint. These stressors were administered intermittently for 6 weeks.

All groups underwent a four-week intervention treatment, with body weights recorded weekly. PCPA injections were administered for the first 2 days of each week to sustain the insomnia model. During the chronic unpredictable mild stress (CUMS) period, SZRD treatment commenced in the third week and continued through the sixth week. Following behavioral testing, fecal samples were collected directly from the rats using sterile centrifuge tubes and immediately preserved in liquid nitrogen. Subsequently, food was withheld for 12 h while water remained available *ad libitum*. The rats were then anesthetized for blood collection, and thymus and spleen tissues were isolated. Blood samples were allowed to clot at room temperature for 30 min before being centrifuged at 3,500 rpm and 4°C for 15 min. The resulting plasma was collected and stored in cryogenic tubes. Immediately post-euthanasia, brain tissue was harvested. The left hippocampus was quickly dissected on ice for hematoxylin and eosin (H&E) staining (*n* = 3 per group), while the right hippocampus was prepared for metabolomics analysis (*n* = 8 per group). Both plasma and remaining brain tissue samples were stored at −80°C.

### 2.5 Behavioral tests

To evaluate the effects of insomnia, depression, and SZRD supplementation on rat behaviors, the Open Field Test (OFT), Forced Swimming Test (FST), Sucrose Preference Test (SPT), and Tail Suspension Test (TST) were conducted. Before the formal tests, a preliminary experiment was conducted with the rats in each group to acclimate them to the test environment and reduce within-group error. The protocols for these tests were based on established methodologies (Kraeuter et al., 2019; Markov, 2022; Ueno et al., 2022; Alamri et al., 2023).

#### 2.5.1 OFT

The OFT assesses locomotor activity and anxiety-related behaviors in rats. Each rat was placed in the center of an open field box with black walls and an open top. An infrared camera mounted above the box recorded the rat’s activities in an environment with controlled lighting and minimal noise. Following a 2-min adaptation period, the rats were observed for 5 min. The following parameters were recorded: time spent in the central area, total distance traveled, and the number of stances. To prevent olfactory influences, the open field apparatus was cleaned with a low-concentration ethanol solution before each test.

#### 2.5.2 FST

The FST measures behavioral despair in rats. The day before the test, rats underwent a 15-min swimming training session in a water container (25 cm wide, 45 cm high) filled to a depth of 30 cm with water at 25°C. During the formal test, each rat was placed in the same container for a 2-min adaptation period, followed by a 4-min test period. The duration of immobility was recorded, with immobility defined as floating with minimal movements necessary to keep the head above water, interspersed with brief periods of struggling.

#### 2.5.3 SPT

The SPT evaluates anhedonia in rats. Prior to the test, rats underwent a 48-h training period where they were provided with both 2% sucrose solution and regular drinking water in two bottles placed on opposite sides of the cage, with the positions of the bottles switched every 8 h. Following the training period, food and water were withheld for 24 h. During the test, rats were given access to the 2% sucrose solution and regular water for 1 h. The volumes of sucrose solution and water consumed were measured before and after the test, and the sucrose preference was calculated using the formula: Sucrose preference (%) = (sucrose consumption/(sucrose consumption + water consumption)) × 100%.

#### 2.5.4 TST

The TST assesses behavioral despair in rats. Each rat’s tail was secured with medical tape 2 cm from the base and attached to a horizontal rod positioned approximately 50 cm above the surface of the experimental table. The rat was suspended with its head oriented downward, approximately 5 cm above the table surface. After a 2-min adaptation period, the rat’s behavior was observed, and the duration of immobility was recorded over a 4-min period. Immobility was defined as the absence of any significant movement, except for the minimal necessary movements to maintain balance.

### 2.6 H&E staining and ELISA analysis

Hippocampal tissue samples were fixed in 4% paraformaldehyde (Leagene, 0201A23, China), followed by paraffin embedding and sectioning. The sections of the hippocampal CA1 region were stained with hematoxylin and eosin (H&E) staining solution (Baso, China). Histological changes were observed and documented using a light microscope.

The remaining brain tissues, including the hypothalamus, were thawed at 4°C. Neurotransmitter and amino acid levels, specifically 5-hydroxytryptamine (5-HT), norepinephrine (NE), dopamine (DA), gamma-aminobutyric acid (GABA), and glutamic acid (Glu), were quantified using commercially available rat ELISA kits (Bioswamp, China) following the manufacturer’s instructions.

### 2.7 16S rRNA gene sequencing

Fecal samples were collected from the rats after the final SZRD treatment and stored at −80°C. Total community genomic DNA was extracted using the E.Z.N.A™ Mag-Bind Soil DNA Kit (Omega, M5635-02, United States) in accordance with the manufacturer’s protocol. DNA concentrations were measured using the Qubit dsDNA HS Assay Kit (ThermoFisher, Q32854, United States) to ensure sufficient amounts of high-quality genomic DNA were obtained. The 16S rRNA V3–V4 region was amplified using the 2 × Hieff^®^ Robust PCR Master Mix (Yeasen, 10105ES03, China) and two universal bacterial 16S rRNA gene primers: forward primer (CCTACGGGNGGCWGCAG) and reverse primer (GACTACHVGGGTATCTAATCC). PCR amplification was conducted in a thermal cycler (Applied Biosystems 9700, United States) with the following conditions: initial denaturation at 95°C for 3 min, 5 cycles of denaturation at 95°C for 30 s, annealing at 45°C for 30 s, and elongation at 72°C for 30 s, followed by 20 cycles of denaturation at 95°C for 30 s, annealing at 55°C for 30 s, and elongation at 72°C for 30 s, and a final extension at 72°C for 5 min.

Amplicon products were purified using Hieff NGSTM DNA Selection Beads (Yeasen, 12601ES56, China) to remove free primers and primer-dimer species. The purified amplicons from each reaction mixture were pooled in equimolar ratios based on their concentrations. Sequencing was performed on the Illumina MiSeq platform (Illumina, United States) according to the manufacturer’s instructions. Effective tags were clustered into operational taxonomic units (OTUs) at a similarity threshold of ≥97% using Usearch software (version 11.0.667). Bacterial and fungal OTU representative sequences were classified taxonomically by aligning them against the RDP and UNITE fungal ITS databases. *α*-diversity indices, including Chao1, Ace, Shannon, and Simpson indices, were calculated to evaluate the richness and diversity of the microbial communities. *β*-diversity, which assesses differences in microbial community composition between samples, was visualized using dimensional reduction techniques such as principal coordinate analysis (PCoA) and non-metric multidimensional scaling (NMDS). Linear discriminant analysis (LDA) Effect Size (LEfSe) was employed to identify gut microbiome features with significant differences in abundance between groups.

### 2.8 Plasma and hippocampal metabolomics studies

Plasma samples were thawed, and 50 μL was carefully transferred into a 1.5 mL microcentrifuge tube. To this, 140 μL of cold methanol and 10 μL of heptadecanoic acid (0.5 mg/mL) were added. The mixture was vortexed for 60 s and ultrasonicated in an ice water bath for 5 min. Subsequently, the mixture was cooled at −20°C for 10 min and then centrifuged at 14,000 rpm at 4°C for 10 min. A 200 μL aliquot of the supernatant was dried using a vacuum centrifugal concentrator. To the dried sample, 30 μL of methoxamine hydrochloride solution (15 mg/mL) was added, followed by vortexing for 60 s and incubation at 40°C for 1 h. Then, 30 μL of N,O-bis(trimethylsilyl)trifluoroacetamide (BSTFA) with 1% trimethylchlorosilane (TMCS) reagent (Macklin, C14859253, China) was added, and the mixture was reacted at 70°C for 1.5 h. Finally, the mixture was transferred to a sample vial for GC–MS analysis. For hippocampal tissue, after thawing, the samples were placed in a glass homogenizer with nine times their volume of ultrapure water. The mixture was homogenized on ice to obtain a hippocampal tissue homogenate. The preparation of hippocampal tissue sample solutions followed the same procedure as that for plasma samples. Quality control (QC) samples were prepared by pooling equal aliquots (5 μL) from each sample. Before formal analysis, QC samples were analyzed continuously six times to ensure reproducibility. To evaluate the precision of the GC–MS method across different batches, a QC sample was analyzed after every 10 samples. GC–MS analysis was conducted using the 7890B-5977B GC–MS system (Agilent Technologies, Santa Clara, CA, United States) equipped with an HP-5 ms UI capillary column (30 m × 0.25 mm × 0.25 μm, Agilent J&W Scientific, Folsom, CA, United States). The temperature program was as follows: the initial temperature was 70°C, increased at 20°C/min to 160°C and held for 1 min, then increased at 30°C/min to 250°C and held for 1 min, and finally increased at 20°C/min to 300°C and held for 4 min. The carrier gas, high-purity helium, was maintained at a flow rate of 1.0 mL/min, with an injection volume of 1 μL and a split ratio of 10:1. The mass spectrometer (MS) scanned in the mass range of 50–500 m/z. The inlet temperature was set at 260°C, and the temperatures for the mass selective detector (MSD) transfer line, ion source, and quadrupole were 280, 230, and 150°C, respectively. The electron impact (EI) ion source operated at an electron energy of 70 eV. Data were collected in scan mode.

The raw GC–MS data were processed using Analysis Base File Converter software to convert the format. Subsequently, the data were standardized using MS-DIAL v4.70 software. Compound identification was performed using MS-DIAL software and the NIST 14.0 standard mass spectrometry database. Principal component analysis (PCA) was employed to reduce the data set to principal component 1 (PC1) and PC2, with PC1 representing the component with the highest variance. The Mann–Whitney U test was used to analyze two non-parametric data sets (Kato et al., 2018). The false discovery rate (FDR) was calculated to reduce the rate of false positives, and differential metabolites were identified based on a fold change (FC) value with FDR < 0.05 and log_2_FC > 1 or < −1.

### 2.9 Analysis of SCFAs

SCFAs in fecal samples were analyzed using GC–MS. Fecal samples were collected from the rats and prepared for analysis. Chromatographic separation was achieved using an HP-FFAP capillary column (30 m × 0.25 mm × 0.25 μm, Agilent J&W Scientific, United States). The temperature program was set as follows: the initial temperature was maintained at 90°C, then increased at a rate of 10°C/min to 150°C and held for 0.5 min; subsequently, it was increased at a rate of 10°C/min to 160°C and held for 0.5 min; finally, it was increased at a rate of 10°C/min to 180°C and held for 1 min. Other GC–MS instrument parameters were consistent with those detailed in the “Plasma and hippocampal metabolomics studies” section. Data acquisition was performed in selected ion monitoring mode, and the analysis was carried out using MassHunter quantitative analysis software (Agilent Technologies).

### 2.10 Correlation analysis

Spearman correlation analysis was employed to investigate the relationships among neurotransmitters, microbiota, and metabolites. This analysis was performed using R version 4.3.1 to calculate the correlation coefficients (*r*) and corresponding *p*-values. Nodes in the correlation network were selected based on the criteria of |r| ≥ 0.6 and *p*-value <0.05. Network edges and weights were computed using the “igraph” package in R. The nodes and edges data were then imported into Gephi version 0.10.1 to construct a correlation network graph. Nodes were ranked according to degree, weighted degree, and betweenness centrality to assess their relative importance within the network.

### 2.11 Statistical analysis

The measurement data were analyzed using GraphPad Prism 8.0 software and expressed as mean ± standard deviation (SD). Initially, the Shapiro–Wilk test was employed to assess normality, followed by Bartlett’s test for homogeneity of variances. When data conformed to a normal distribution and exhibited homogeneity of variance, one-way ANOVA was used to compare the mean differences between groups, with Tukey’s multiple comparisons test employed for post-hoc analysis. If the data conformed to a normal distribution but exhibited heterogeneity of variance, the Brown-Forsythe and Welch ANOVA were used, followed by Dunnett’s T3 multiple comparisons test for post-hoc analysis. For data that did not conform to a normal distribution, the Kruskal-Wallis test was applied to compare mean differences between groups, with Dunn’s multiple comparisons test used for post-hoc analysis. Statistical significance was defined as *p*-values <0.05.

## 3 Results

### 3.1 The chemical components of SZRD

**Figure 1:**
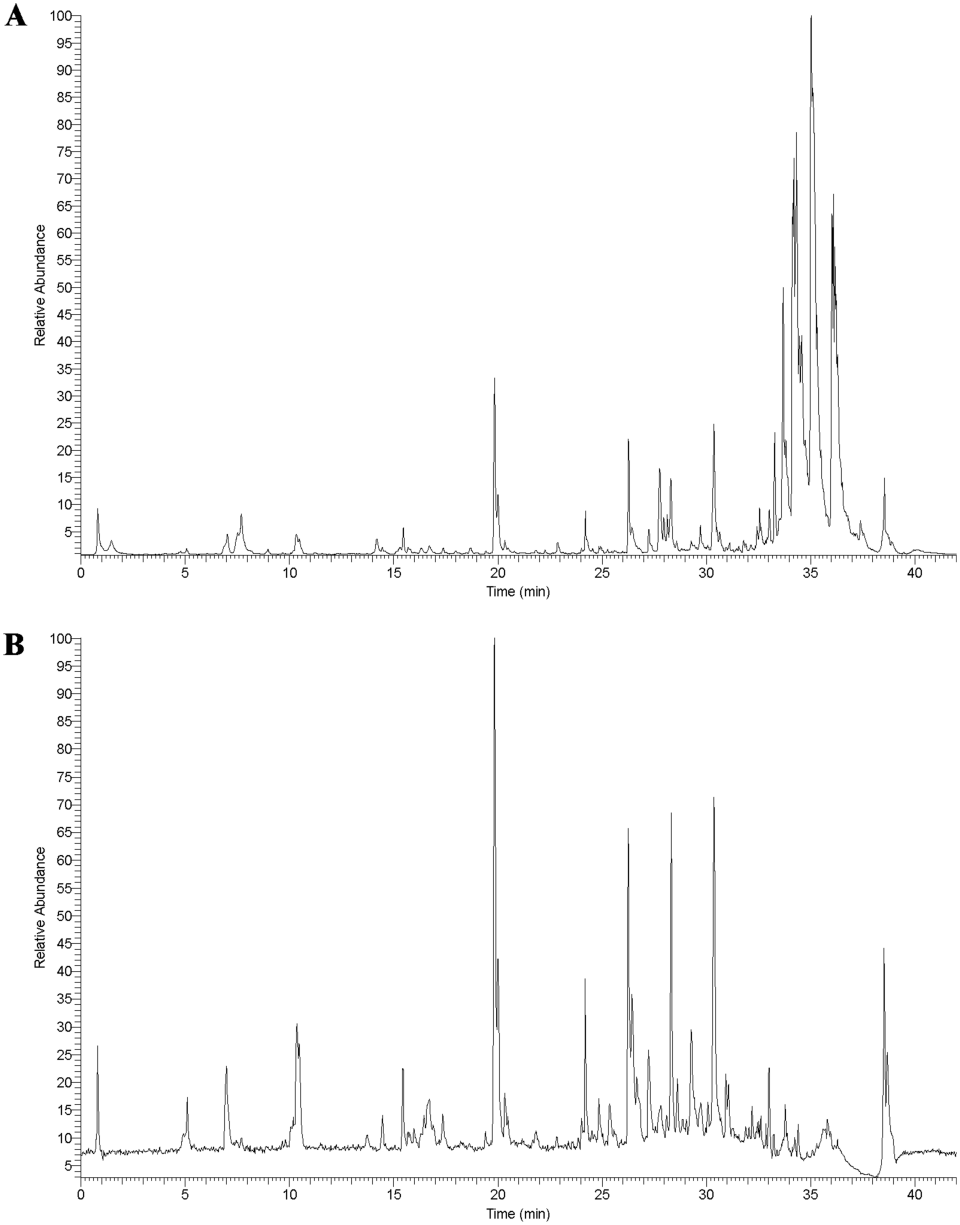
The total ion chromatogram of SZRD in POS (A) and NEG (B) mode.

To identify the chemical components in SZRD, we performed ultra-high-performance liquid chromatography coupled with LTQ-Orbitrap mass spectrometry (UHPLC-LTQ-Orbitrap-MS) analysis. The characteristic peaks in both positive and negative ion modes are shown in Figure 1. Using Compound Xcalibur 4.1 software and literature comparisons, we determined the retention times, molecular formulas, exact molecular weights, and ionization modes for each compound, leading to the identification of 66 chemical components in SZRD (Supplementary Table S1). Among these, flavonoids were the most abundant, with 34 distinct types identified. Additionally, we identified triterpenes, phthalides, steroids, alkaloids, organic acids, and phenols. Our results indicate that flavonoid compounds, such as liquiritin, mangiferin, liquiritin apioside, and formononetin, are the primary active components of SZRD.

### 3.2 Behavioral characteristics of insomnia and depression rat models

**Figure 2:**
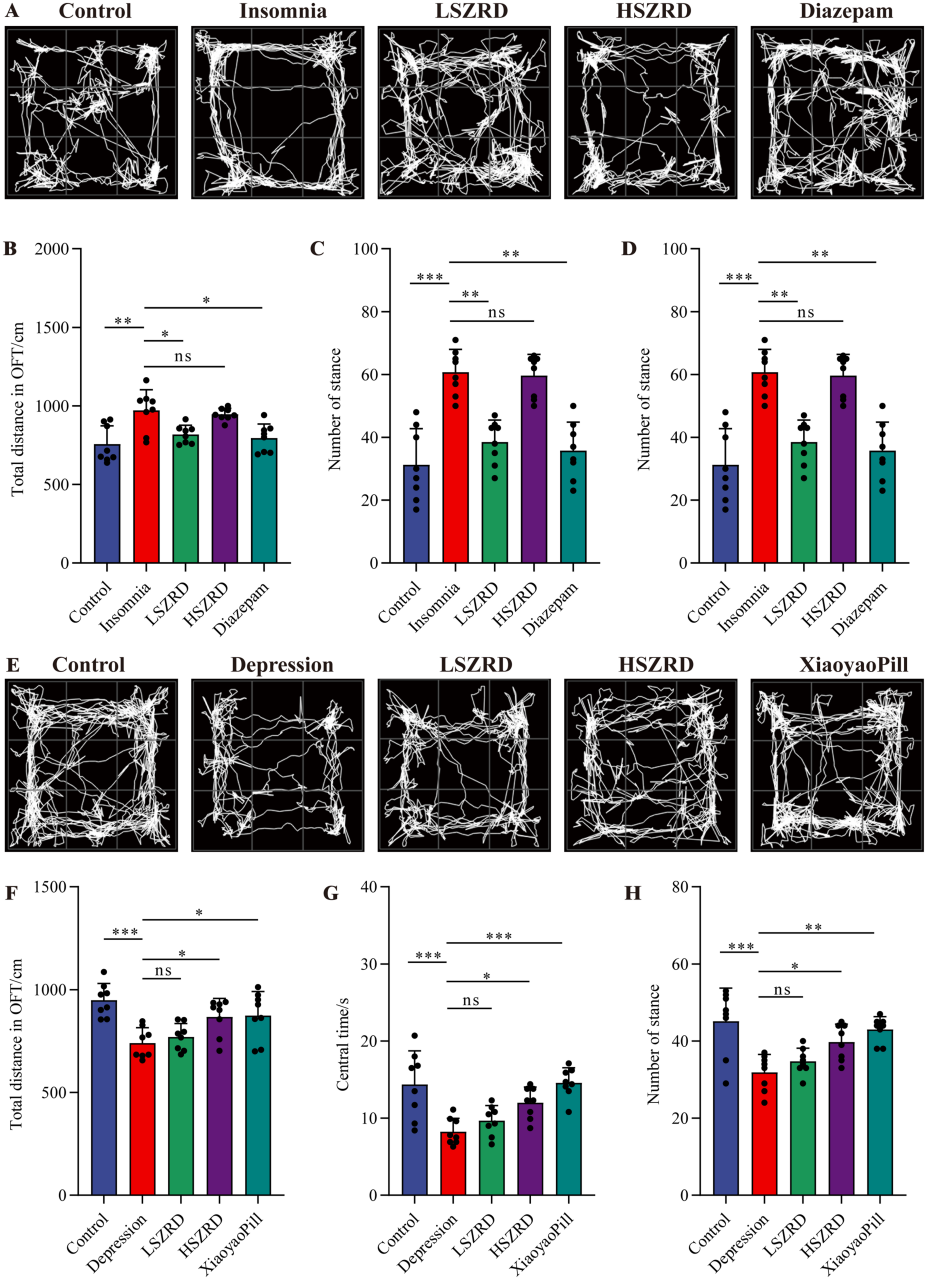
The effects of SZRD on activity in the rat models of insomnia and depression in the OFT. (A) Representative track plots of Control, Insomnia, LSZRD, HSZRD, and Diazepam groups captured by the OFT-100, an open-field activity experimental system for rats. (B) The distance of movement in the open-field box. (C) The central time. (D) The number of stances. (E) Representative track plots of Control, Depression, LSZRD, HSZRD, and XiaoyaoPill groups. (F) Movement distance. (G) The central time. (H) The number of stances. ^*^p < 0.05, ^**^p < 0.01, ^***^p < 0.001, ns: no significant difference, *n* = 8.

**Figure 3:**
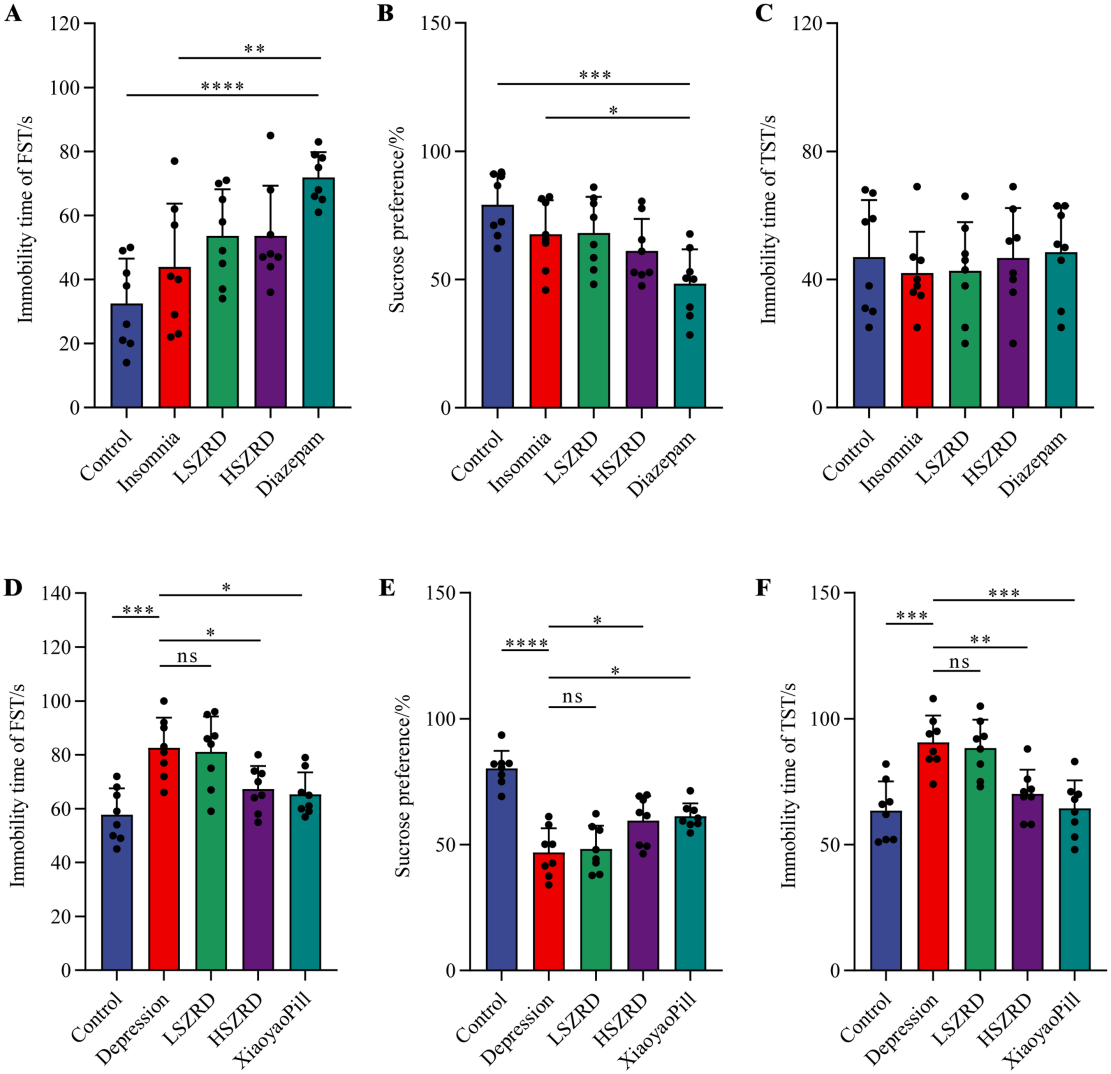
Effects of SZRD on depression-like behaviors in the rat models of insomnia and depression in the FST, SPT, and TST. (A–C) Forced swimming immobility time, sucrose preference, and tail suspension immobility time in the Control, Insomnia, LSZRD, HSZRD, and Diazepam groups. (D–F) Forced swimming immobility time, sucrose preference, and tail suspension immobility time in the Control, Depression, LSZRD, HSZRD, and XiaoyaoPill groups. ^*^p < 0.05, ^**^p < 0.01, ^***^p < 0.001, ^****^p < 0.0001, ns: no significant difference, *n* = 8.

To investigate the impact of SZRD on the behavioral characteristics of insomnia and depression in rats, we conducted the OFT, FST, SPT, and TST. In the OFT, insomnia rats displayed significantly increased activity compared to the Control group, with greater movement distances and more frequent stances within the open field box (Figures 2A,B,D). These rats also showed a marked reduction in central time and increased activity in the peripheral area (Figure 2C). LSZRD supplementation effectively ameliorated these behaviors, whereas no statistically significant difference was observed between the HSZRD and Insomnia groups. Conversely, depression rat models exhibited reduced activity in the open field box, characterized by shorter movement distances and fewer stances compared to the Control group, along with reduced central time (Figures 2E–H). The HSZRD group, however, showed increased activity in the OFT. In the FST, SPT, and TST, insomnia rats treated with diazepam exhibited lower sucrose preference and higher immobility time during swimming. No significant differences were observed among the other groups in these tests (Figures 3A–C), indicating that insomnia rats did not exhibit significant changes in depression-like behaviors, and long-term diazepam administration could induce specific depression-like behaviors. Rat models of depression, in both the FST and TST, demonstrated significant depression-like behaviors, with increased immobility times (Figures 3D,F). Additionally, compared to the Control group, these rats showed reduced sucrose preference in the SPT (Figure 3E). The HSZRD group exhibited significantly reduced immobility times in both the FST and TST, and increased sucrose preference in the SPT, indicating an improvement in depression-like behaviors. No significant differences were observed between the LSZRD and Depression groups in these behavioral tests. These findings suggest that LSZRD can alleviate the behavioral characteristics of insomnia in rats, while HSZRD significantly improves depression in rats.

### 3.3 SZRD reduced pathological changes and neurotransmitter dysregulation in insomnia and depression rats

**Figure 4:**
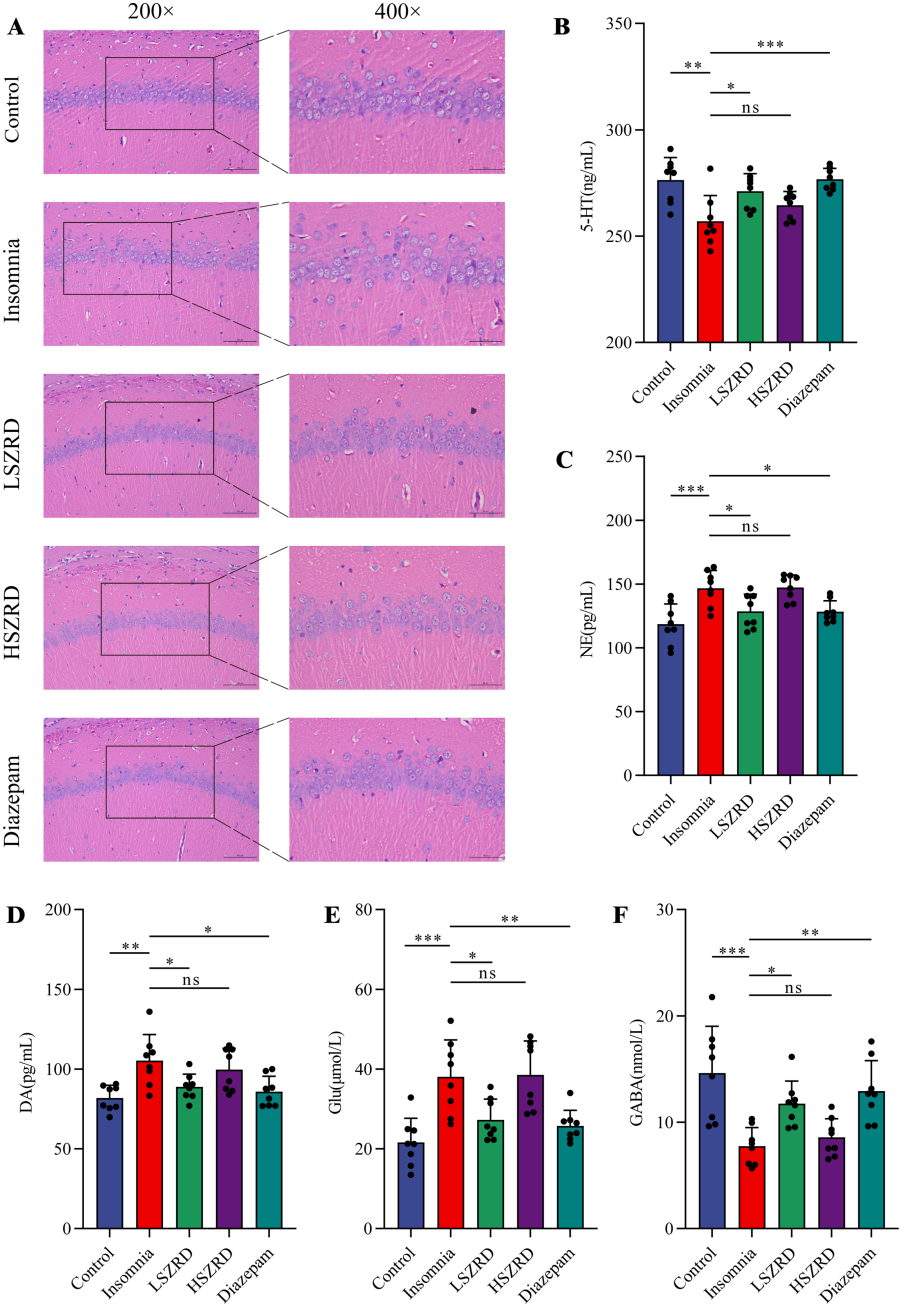
SZRD partially reduces pathological changes and neurotransmitter dysregulation in insomnia rats. (A) Pathological changes in the hippocampal CA1 region in the Control, Insomnia, LSZRD, HSZRD, and Diazepam groups (*n* = 3; the scale of 200× = 100 μm; the scale of 400× = 50 μm). (B–F) Levels of 5-HT, NE, DA, Glu, and GABA. ^*^p < 0.05, ^**^p < 0.01, ^***^p < 0.001, ns: no significant difference, *n* = 8.

**Figure 5:**
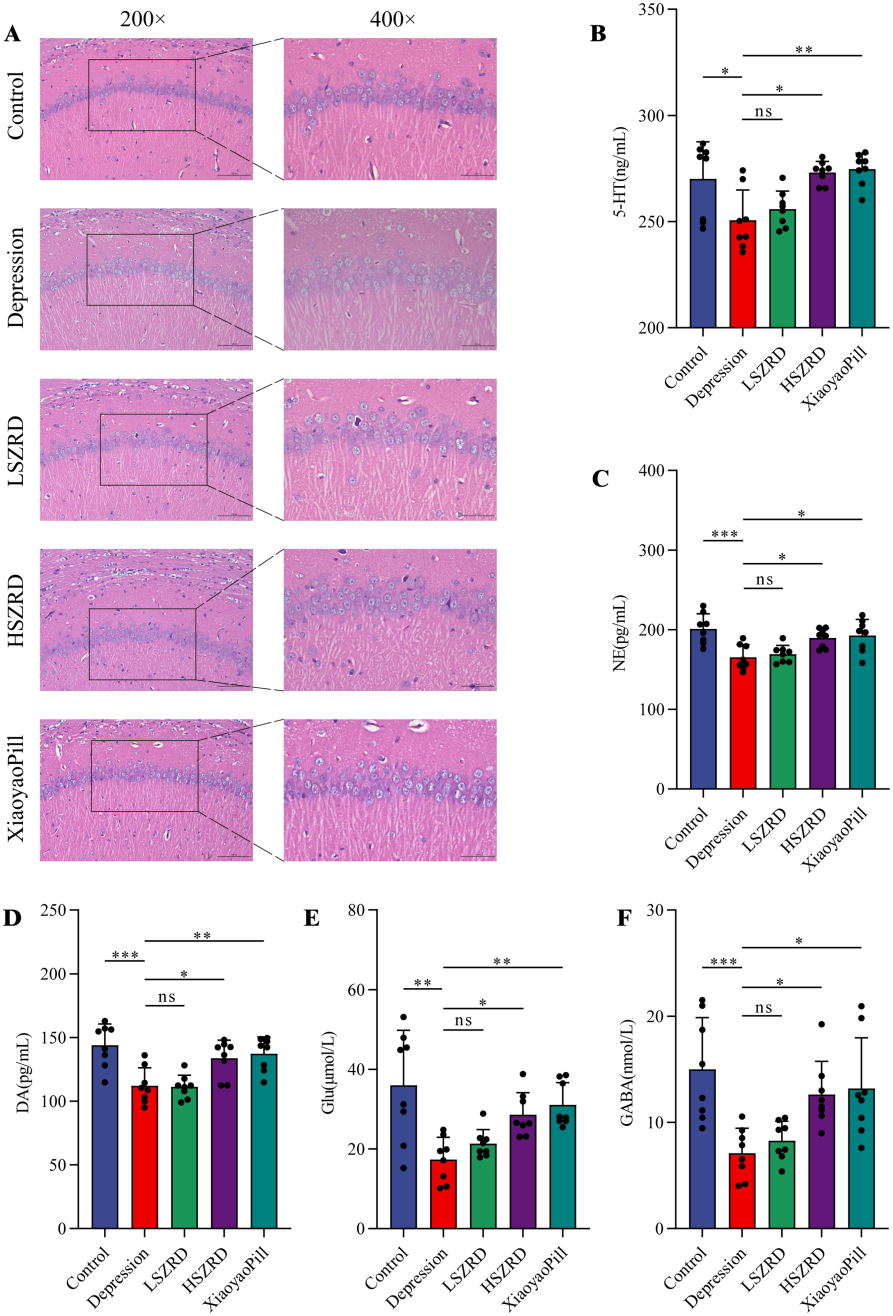
SZRD partially reduces pathological changes and neurotransmitter dysregulation in the rat model of depression. (A) Pathological changes in the hippocampal CA1 region in the Control, Depression, LSZRD, HSZRD, and XiaoyaoPill groups (*n* = 3; the scale of 200× = 100 μm; the scale of 400× = 50 μm). (B–F) Levels of 5-HT, NE, DA, Glu, and GABA. ^*^p < 0.05, ^**^p < 0.01, ^***^p < 0.001, ns: no significant difference, *n* = 8.

Body weight differences were measured weekly (Supplementary Figures S1A, S2A). Organ index analysis revealed statistically significant differences only between the Depression and Control groups (Supplementary Figures S1B,C, S2B,C). The hippocampus, a crucial region for learning, memory, and emotional regulation, is closely associated with insomnia and depression. According to the HE staining of the hippocampal CA1 region, the pyramidal cells of control group were regularly shaped and arranged neatly and densely. Their nuclei were uniformly light blue, and the cytoplasm was abundant, with no neuroglial cell proliferation. Conversely, in the Insomnia and Depression groups, pyramidal cells were disorganized, with many cells showing slight denaturation and sparse cytoplasm. Cell outlines were indistinct, and the boundaries between the nucleus and cytoplasm were poorly defined. Following SZRD treatment, the hippocampal pyramidal cells appeared more orderly, forming 2–3 layers with distinct nuclei (Figures 4A, 5A). These findings indicate that SZRD can ameliorate pathological changes in the hippocampal CA1 region of the rat models of insomnia and depression. Insomnia and depression may cause disturbances in monoamine neurotransmitters (5-HT, NE, and DA) and amino acid neurotransmitters (GABA and Glu) (Lai et al., 2023). In the Insomnia and Depression groups, levels of 5-HT and GABA were lower than in the Control group (Figures 4B,F, 5B,F). Levels of NE, DA, and Glu differed between insomnia and depression (Figures 4C–E, 5C–E). Specifically, these neurotransmitters were elevated in insomnia rats’ brain tissues but decreased in the rat model of depression. Compared to the Insomnia group, the LSZRD group adjusted these neurotransmitter levels, while no statistical differences were observed in the HSZRD group. Conversely, in the Depression group, these neurotransmitters were restored in the HSZRD group, with no statistical differences in the LSZRD group. Thus, SZRD supplementation can partially improve hippocampal pathological changes and regulate neurotransmitter levels in brain tissue, with LSZRD being more effective for insomnia rats and HSZRD for depression rats.

### 3.4 SZRD partially ameliorates gut microbiome disorders in the rat models of insomnia and depression

**Figure 6:**
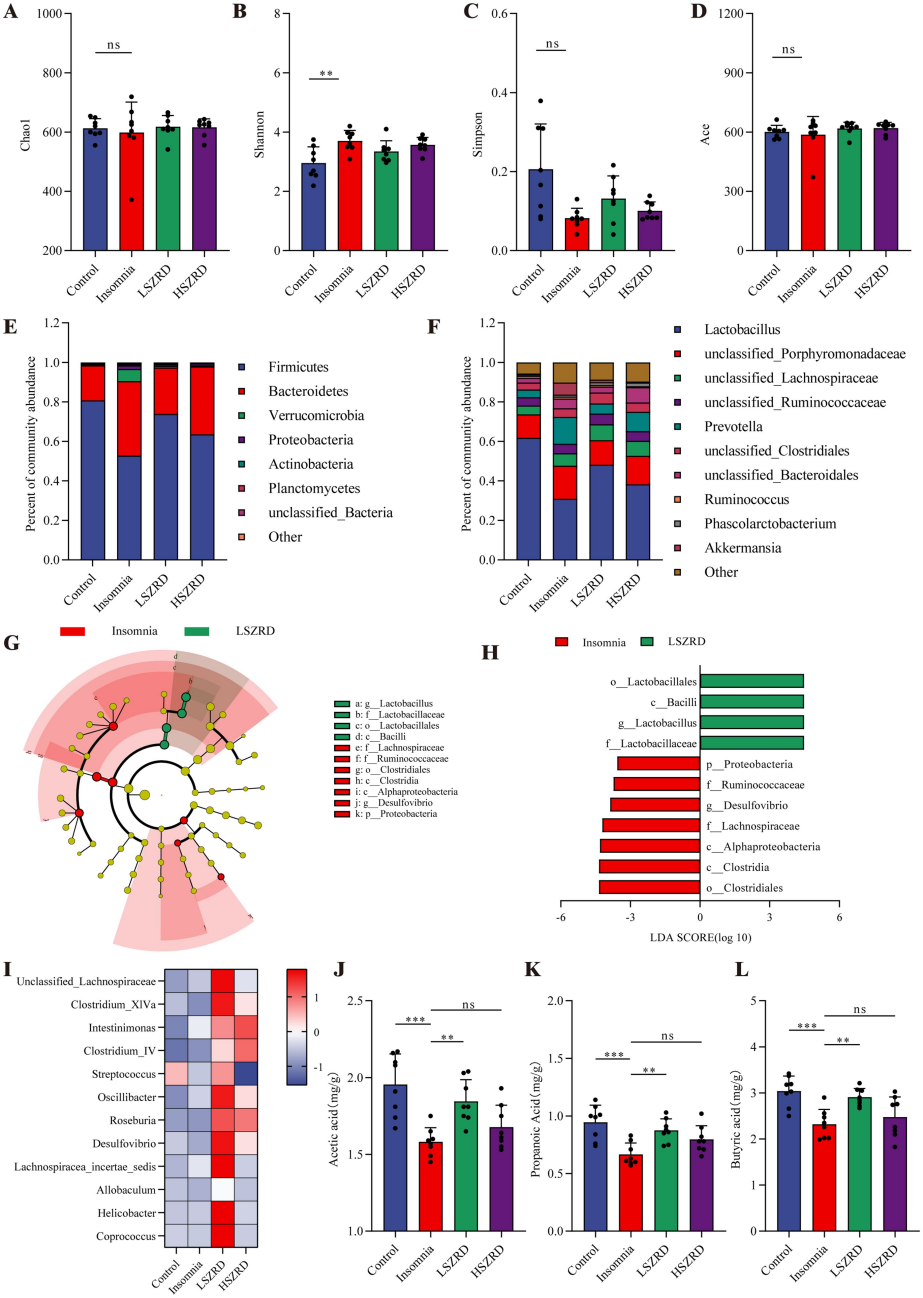
SZRD partially ameliorated gut microbiome disorders in insomnia rats. (A) Chao1 index. (B) Shannon index. (C) Simpson index. (D) Ace index. (E) Microbial population distribution at the phylum level. (F) Microbial population distribution at the genus level. (G) LEfSe analysis of Insomnia and LSZRD groups. (H) LDA histogram of the Insomnia and LSZRD groups. (I) Heatmap of relative abundance of SCFA-producing genera. Color intensity (blue to red) in the heatmap indicates the standardized abundance score for each genus. (J–L) Levels of acetic, propionic, and butyric acids in fecal samples. ^**^p < 0.01, ^***^p < 0.001, ns: no significant difference, *n* = 8.

**Figure 7:**
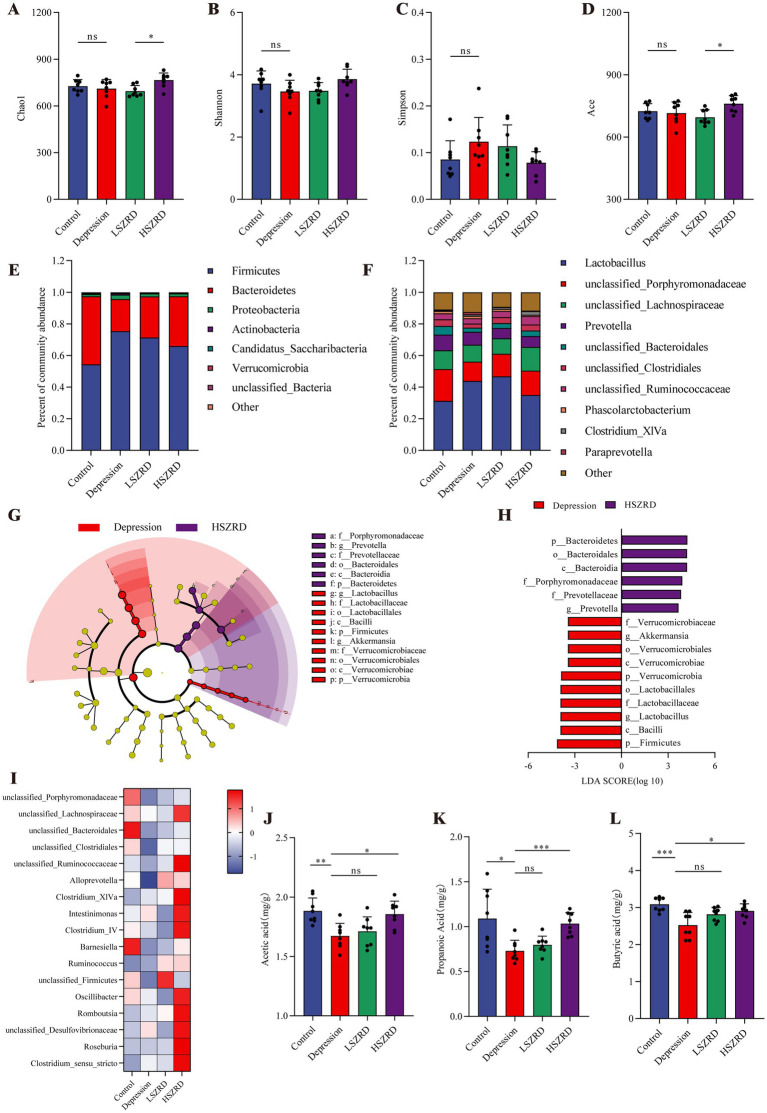
SZRD partially ameliorated gut microbiome disorders in depressed rats. (A) Chao1 index. (B) Shannon index. (C) Simpson index. (D) Ace index. (E) Microbial population distribution at the phylum level. (F) Microbial population distribution at the genus level. (G) LEfSe analysis of Depression and HSZRD groups. (H) LDA histogram of the Depression and HSZRD groups. (I) Heatmap of relative abundance of SCFA-producing genera. (J–L) Levels of acetic, propionic, and butyric acids in fecal samples. ^*^p < 0.05, ^**^p < 0.01, ^***^p < 0.001, ns: no significant difference, *n* = 8.

Research has shown that the gut microbiome is disrupted during periods of insomnia and depression (Sharon et al., 2016). To investigate this, we analyzed fecal samples from each group using 16S rRNA sequencing. In the *α*-diversity analysis, there were no significant differences in the Chao1, Simpson, and Ace indices between the Insomnia group and other groups (Figures 6A,C,D). However, the Insomnia group exhibited a higher Shannon index, indicating increased species richness compared to the Control group (Figure 6B). The ratio of Firmicutes to Bacteroidetes, a commonly used indicator of gut microbiome changes associated with various pathological states including insomnia and chronic stress (Bowers et al., 2020), was significantly reduced in insomnia rats. The Insomnia group showed about a 30% reduction in Firmicutes abundance, accompanied by a corresponding increase in Bacteroidetes (Figure 6E; Supplementary Figure S3A). At the genus level, insomnia rats had reduced abundances of *Lactobacillus*, *Clostridium_XlVa*, *Clostridium_IV*, and *Roseburia*, and increased levels of *Prevotella*, *Phascolarctobacterium*, *Bacteroides*, and *unclassified_Lachnospiraceae* (Figure 6F). The analysis of *β*-diversity, using PCoA and NMDS plots, demonstrated differences in the composition of the gut microbiome between the Insomnia and the Control group. Following SZRD treatment, the LSZRD group separated from the Insomnia group, whereas the HSZRD group remained closer to the Insomnia group (Supplementary Figures S3B,C). For the rat models of depression, there were no significant differences in the four α-diversity indices (Figures 7A–D). However, the Firmicutes to Bacteroidetes ratio was significantly higher in the Depression group, with a 20% increase in Firmicutes and a corresponding decrease in Bacteroidetes (Figure 7E; Supplementary Figure S4A). At the genus level, rat models of depression showed decreased abundances of *unclassified_Porphyromonadaceae*, *Prevotella*, *Alloprevotella*, and *Clostridium_IV*, and increased levels of *Lactobacillus*, *Alistipes*, *Desulfovibrio*, and *Bifidobacterium* (Figure 7F). β-Diversity analysis showed separation between the Control and Depression groups, with the HSZRD group closer to the Control group compared to the LSZRD group (Supplementary Figures S4B,C).

To investigate the impact of LSZRD on the gut microbiome in insomnia rats, LEfSe analysis was conducted between the Control and Insomnia groups, as well as between the Insomnia and LSZRD groups. The Insomnia group had an increased abundance of Bacteroidetes and its lower taxa (LDA > 3.0, Supplementary Figures S3D,E). LSZRD supplementation increased the abundance of Firmicutes and its lower taxa, including *o_Lactobacillales*, *c_Bacilli*, *g_Lactobacillus*, and *f_Lactobacillaceae* (LDA > 3.0, Figures 6G,H). Additionally, LSZRD increased the abundance of SCFA-producing genera such as *Clostridium_XlVa*, *Clostridium_IV*, *Roseburia*, *Intestinimonas*, and *Coprococcus* (Figure 6I). These genera are associated with the production of SCFAs, including acetic, propionic, and butyric acids (Zhuang et al., 2021). GC–MS analysis of fecal samples showed reduced SCFA levels in insomnia rats, which were increased by LSZRD supplementation. The HSZRD group did not show a significant difference compared to the Insomnia group (Figures 6J–L), suggesting that LSZRD can partially restore gut microbiome balance in insomnia rats. Similarly, in the depressed rats, LEfSe analysis revealed the enrichment of Firmicutes and its lower taxa in the Depression group (LDA > 3.0, Supplementary Figures S4D,E). HSZRD supplementation increased the abundance of Bacteroidetes and its lower taxa, including *o_Bacteroidales*, *c_Bacteroidia*, *f_Porphyromonadaceae*, *f_Prevotellaceae*, and *g_Prevotella* (LDA > 3.0, Figures 7G,H). The SCFA-producing genera, such as *unclassified_Lachnospiraceae*, *Clostridium_XlVa*, *Intestinimonas*, *Clostridium_IV*, and *Roseburia*, were also enriched in the HSZRD group (Figure 7I). GC–MS analysis confirmed reduced SCFA levels in the Depression group compared to the Control group, with HSZRD supplementation reversing this reduction. No significant difference was observed in the LSZRD group compared to the Depression group (Figures 7J–L). These findings suggest that LSZRD supplementation partially improves the gut microbiome composition in insomnia rats, while HSZRD supplementation is more effective in depressed rats.

### 3.5 SZRD improves metabolic changes in insomnia and depression rats

**Figure 8:**
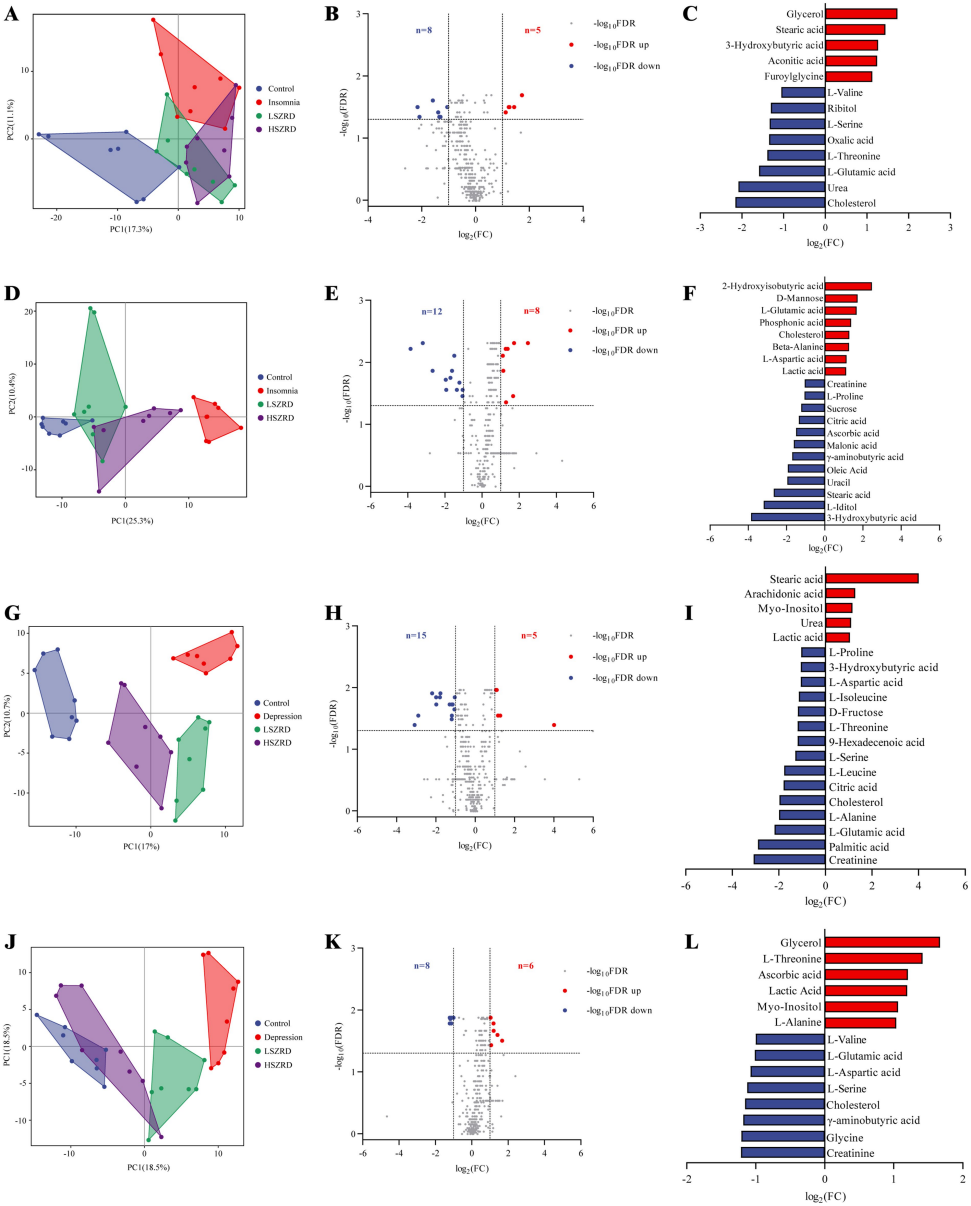
SZRD improved metabolic changes in the rat models of insomnia and depression. Plasma metabolomics analysis in insomnia rats (A–C). (A) PCA plot of Control, Insomnia, LSZRD, and HSZRD groups (*n* = 8). (B) Volcano plot of Control and Insomnia groups. (C) The histogram of differential metabolites highlights significantly upregulated metabolites in red and downregulated metabolites in blue. Hippocampus metabolomics analysis in insomnia rats (D–F). (D) PCA plot (*n* = 8). (E) Volcano plot of Control and Insomnia groups. (F) Differential metabolites histogram. Plasma metabolomics analysis in depression rats (G–I). (G) PCA plot (*n* = 8). (H) Volcano plot of Control and Depression groups. (I) Differential metabolites histogram. Hippocampus metabolomics analysis in depressed rats (J–L). (J) PCA plot (*n* = 8). (K) Volcano plot of Control and Depression groups. (L) Differential metabolites histogram.

After observing that SZRD supplementation partially ameliorated gut microbiome disorders in the rats, we examined the metabolic profiles of plasma and hippocampal tissues using (GC–MS). The superimposed chromatograms of the QC samples demonstrated the repeatability and reliability of the research (Supplementary Figure S5). The PCA plot indicated that the Control group was distinctly separate from the Insomnia group, and the LSZRD group was closer to the Control group than the HSZRD group (Figures 8A,D). A volcano plot analysis for the Control and Insomnia groups identified 13 differential metabolites in the plasma of insomnia rats, including five upregulated and eight downregulated metabolites (Figures 8B,C). In the hippocampus of insomnia rats, 20 differential metabolites were identified, including eight upregulated and 12 downregulated metabolites (Figures 8E,F). Statistical analysis revealed that LSZRD supplementation significantly increased the levels of amino acid metabolites (L-Valine, L-Threonine, and L-Serine) in the plasma of insomnia rats (Supplementary Figure S6A). Additionally, the LSZRD group exhibited elevated levels of *γ*-aminobutyric acid and reduced levels of L-Glutamic acid in the hippocampus (Supplementary Figure S6C). Similarly, the PCA plot indicated that the Control group was distinctly separate from the Depression group, with the HSZRD group being closer to the Control group than the LSZRD group (Figures 8G,J). In the plasma of depressed rats, 20 differential metabolites were identified, including 5 upregulated and 15 downregulated metabolites. In the hippocampus of depressed rats, 20 differential metabolites were identified, including 8 upregulated and 12 downregulated metabolites (Figures 8H,I,K,L). HSZRD supplementation increased the levels of several amino acid metabolites (L-Alanine, L-Aspartic acid, L-Proline, L-Glutamic acid, L-Leucine, L-Serine, L-Isoleucine, and L-Threonine) in the plasma of depressed rats (Supplementary Figure S7A), and levels of *γ*-aminobutyric acid and L-Glutamic acid in the hippocampus (Supplementary Figure S7C). The differential metabolites identified included amino acids, fatty acids, carbohydrates, and organic acids. These metabolites were analyzed using MetaboAnalyst 5.0 for KEGG pathway analysis. Interestingly, the alanine, aspartate, and glutamate metabolism pathway was involved in both insomnia and depression processes (Supplementary Figures S6B,D, S7B,D). We found that levels of amino acid metabolites (L-Valine, L-Threonine, L-Serine, etc.) were reduced in the plasma of the rat models of both insomnia and depression. In the hippocampus, *γ*-aminobutyric acid levels were decreased in both conditions, while L-Glutamic acid levels showed opposing trends. Specifically, the levels of amino acid metabolites were significantly reduced in the plasma of rats with insomnia and depression. The abnormal expression of L-Glutamic acid and GABA could be partially prevented by LSZRD and HSZRD supplementation.

### 3.6 Correlation network of SZRD in intervening the rat models of insomnia and depression

**Figure 9:**
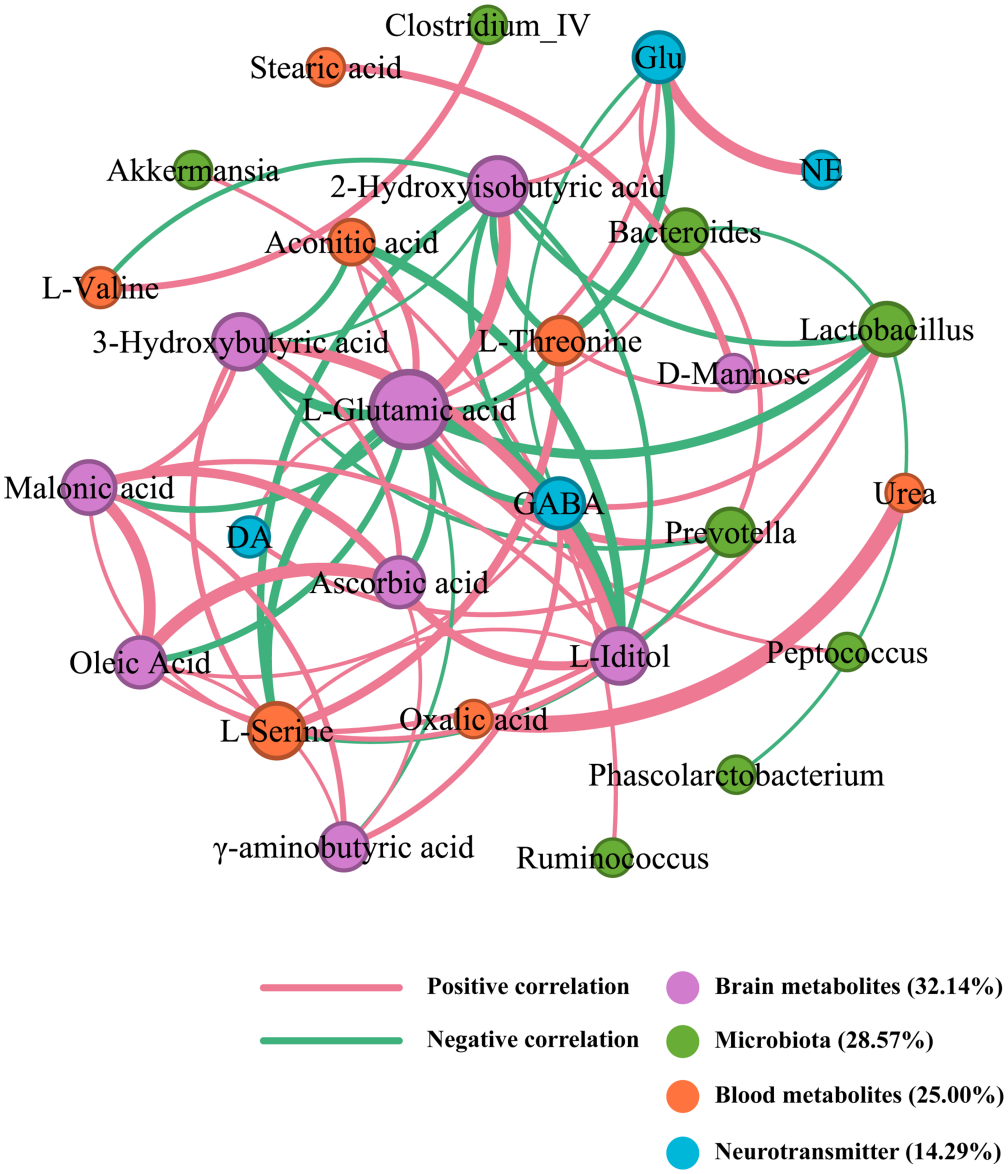
Correlation network of SZRD in intervening insomnia rats.

An integrative network analysis of gut microbiome and metabolome data revealed strong correlations among the gut microbiome, metabolites, and neurotransmitters in the correlation network of SZRD intervention in insomnia rats (Figure 9). The brain metabolite L-Glutamic acid, with a degree value of 16, emerged as the primary contributor within this network. Meanwhile, L-Serine, *Lactobacillus*, and GABA, with degree values of 8, 7, and 6 respectively, were the nodes with the highest degree values among blood metabolites, microbiota, and neurotransmitters (Supplementary Table S2). These findings suggest their significant involvement in the mechanism underlying insomnia. L-Glutamic acid was negatively correlated with *Lactobacillus* and positively correlated with *Bacteroides* and *Prevotella*. Additionally, L-Glutamic acid showed a negative correlation with the brain metabolite *γ*-aminobutyric acid and the blood metabolites L-Serine and L-Threonine. The neurotransmitters Glu and DA were positively correlated with L-Glutamic acid, while GABA was negatively correlated with L-Glutamic acid.

**Figure 10:**
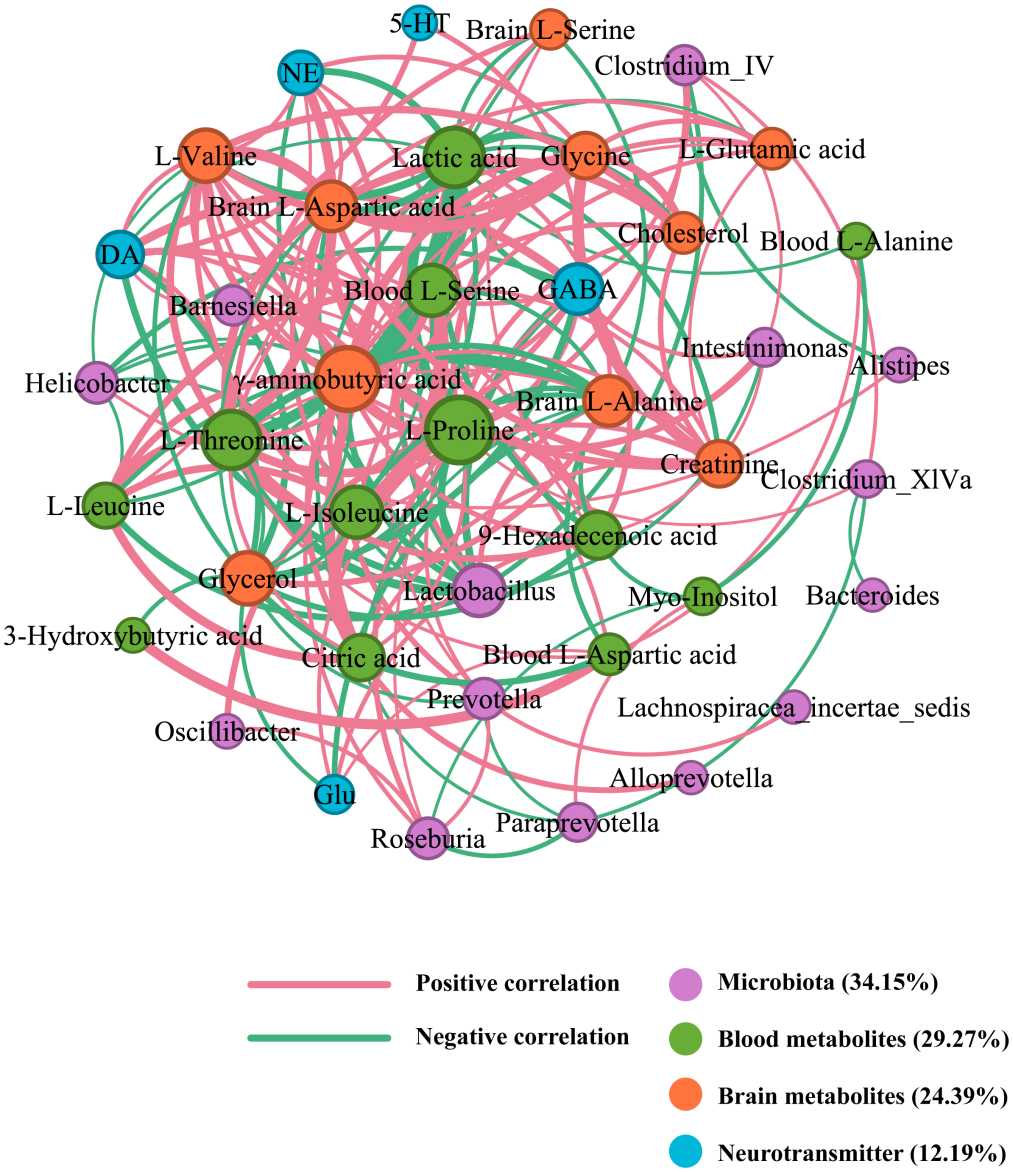
Correlation network of SZRD in intervening depressed rats.

In the correlation network of SZRD intervention in depressed rats, the blood metabolite L-Proline, with a degree value of 26, was identified as the primary contributor (Figure 10). Additionally, γ-aminobutyric acid, *Lactobacillus*, and GABA, with degree values of 24, 15, and 14, respectively, were the most significant nodes among brain metabolites, microbiota, and neurotransmitters (Supplementary Table S3). These elements are crucial in influencing the mechanisms of depression. L-Proline was negatively correlated with *Lactobacillus* and *Helicobacter*, and positively correlated with *Clostridium IV*, *Clostridium XlVa*, *Prevotella*, and *Roseburia*. The neurotransmitters Glu, GABA, DA, and NE were positively correlated with L-Proline. L-Proline also showed positive correlations with the blood metabolites L-Isoleucine and L-Threonine, as well as the brain metabolite γ-aminobutyric acid.

## 4 Discussion

This study demonstrates that different doses of SZRD have positive effects on the gut microbiome and metabolites in rat models of PCPA-induced insomnia and CUMS-induced depression. LSZRD supplementation partially improved the behaviors observed in the OFT for insomnia rats, while HSZRD promoted recovery from depression-like behaviors in depressed rats. Furthermore, SZRD was found to regulate neurotransmitter levels, reduce pathological damage in the hippocampus, restore dysregulation of the gut microbiome, and improve metabolic disorders in plasma and hippocampal tissue. These findings support the conclusion that SZRD intervene in different disease models of insomnia and depression through gut microbiome modulation and metabolomic pathways.

5-HT is a crucial monoamine neurotransmitter affecting emotions, memory, sleep, and temperature regulation. It plays a role in maintaining sleep by interacting with other neurotransmitter pathways (Benton et al., 2022). Tryptophan can be converted to 5-HT with the help of tryptophan hydroxylase and amino acid decarboxylase (Ślifirski et al., 2021). PCPA is commonly used to induce insomnia models by decreasing 5-HT levels through inhibition of tryptophan hydroxylase (Dong et al., 2021). In this study, the insomnia model was reproduced using intraperitoneal injections of PCPA. Insomnia rats did not show significant changes in depression-like behavior tests, but there was a significant decrease in 5-HT levels in brain tissues, indicating the model’s effectiveness and specificity for insomnia without significant depression-like behavior.

The CUMS model effectively simulates human depressive symptoms and a range of emotional expressions (Markov and Novosadova, 2022). Model animals show significant responses to effective medications for mood disorders, and the biological mechanisms producing mood disorders in these animals are similar to those in humans, meeting the criteria for evaluating animal models of mood disorders (Markov, 2022). We used 10 different CUMS stress methods, housed the rats in individual cages, and applied one stress method each day to prepare the depression model. Depressed rats displayed lower mobility in the OFT and significant behavioral despair and pleasure deficits in the FST, SPT, and TST, without significant anxiety-like behavior. Notably, the Diazepam group had a significantly lower sucrose preference and longer immobility time in the FST than the Insomnia group, likely due to long-term diazepam use (Kaur et al., 2016). Overall, SZRD supplementation improved anxiety-like behavior induced by PCPA and depression-like behavior due to CUMS.

The hippocampus, involved in memory, learning, and cognitive functions, also generates sensations, experiences, and associated emotions, playing a crucial role in regulating sleep and emotions (FeldmanHall et al., 2021). Studies on patients with insomnia and depression have shown abnormal local cerebral blood flow in the hippocampal region, as well as functional and structural changes in the hippocampus (Xu et al., 2023). Chronic insomnia and depression may reduce the number of nerve cells in the hippocampus (Chen et al., 2020). Our study found that the hippocampal CA1 region in the rat models of insomnia and depression rats had sparsely arranged and disorganized cells, many showing degeneration and lighter color. After SZRD treatment, cells were more closely arranged and darker, indicating varying degrees of improvement. Our previous study found different expressions of 5-HT, NE, and DA in mice with insomnia and depression (Zhang et al., 2023). Monoamine and amino acid neurotransmitters are involved in the biological processes of neurological disorders, including insomnia and depression. GABA and Glu are the principal inhibitory and excitatory neurotransmitters of the central nervous system, respectively. In our study, neurotransmitter content trends were inconsistent in insomnia and depression rats. NE, DA, and Glu levels decreased in insomnia rats after LSZRD treatment, while 5-HT and GABA levels increased. HSZRD increased neurotransmitter levels in depressed rats. Therefore, SZRD supplementation alleviated pathological changes in the hippocampus and prevented neurotransmitter dysregulation in the brain tissue of rats with insomnia and depression.

Studies in rodent models have shown that the gut microbiome is associated with stress and anxiety (Foster and McVey Neufeld, 2013). Rats receiving gut microbiomes from patients with major depression displayed behavioral deficits in the elevated plus maze (EPM), SPT, and OFT (Kelly et al., 2016). Similarly, reduced microbiome diversity has been found in patients with acute and chronic insomnia (Wang Z. et al., 2022). These results support the association of gut microecological dysbiosis with psychiatric disorders, including insomnia, anxiety, and depression. In our study, the Shannon index increased in insomnia rats, suggesting that PCPA led to an increase in gut microbiome richness without significant changes in species diversity. In contrast to the Control group, there were no significant differences in *α*-diversity indexes of depression rats. However, significant differences were observed in *β*-diversity analysis. Our results are supported by recent research that reviewed the gut microbiome in depression, showing no variation in α-diversity between depression and control groups in over half of the analyses from 137 clinical and 455 preclinical studies (Liu et al., 2022). SZRD supplementation increased the number of SCFA-producing genera in the rat models of insomnia and depression. *Blautia*, producing butyrate (Feng et al., 2023), and *Coprococcus*, producing propionate, were enriched after SZRD treatment (Louis and Flint, 2017). Recent studies indicate that SCFAs could serve as intermediaries connecting the gut microbiome with sleep mechanisms in the brain (Heath et al., 2020). Butyrate treatment suppressed depression-like behaviors in rodents *in vitro* and *in vivo* (Yamawaki et al., 2018). Increased propionic acid levels in feces are associated with longer sleep (Magzal et al., 2021). Reduction of acetate-producing bacteria results in long-term acetate deficiency, associated with decreased synaptophysin (SYP) in the hippocampus, leading to impaired learning and memory (Zheng et al., 2021). SZRD supplementation increased SCFA levels in fecal samples of insomnia and depression rats. Additionally, LSZRD treatment improved the levels of Firmicutes and their taxonomic microbiome in insomnia rats. Interestingly, we observed an increased abundance of *Lactobacillus* in depression rats. While *Lactobacillus* and *Bifidobacterium* are commonly used as probiotic supplements to alleviate depressive phenotypes, they are also enriched in both patients and animal models with depression (Liu et al., 2022). It is crucial to recognize that in mood disorders such as depression, there may not be a clear distinction between beneficial and pathogenic bacteria. Ingestion of *Lactobacillus reuteri* and *Lactobacillus helveticus* has been reported to cause depressive phenotypes and pleasure deficits (Wang et al., 2020) and to disrupt social behaviors (Partrick et al., 2021), suggesting strain-specific differences in the effects of *Lactobacillus*. Therefore, probiotics may pose health hazards when administered inappropriately. Overall, SZRD supplementation partially ameliorated gut microbiome disorders and restored SCFA levels.

Studies have confirmed that metabolic processes are associated with sleep, and depression also leads to changes in specific metabolites (Humer et al., 2020). These metabolites are widely used to identify pathological changes in various diseases due to their essential physiological roles. A metabolomics study integrating animal models of depression suggested that the hippocampus (about 63%) was the most extensively used brain tissue, and plasma (about 40%) was the most commonly used sample type in peripheral tissues (Pu et al., 2021). Therefore, we selected plasma and hippocampal tissues for metabolomics studies and found that amino acid metabolites (L-Valine, L-Threonine, L-Serine, etc.) displayed a down-regulation trend in the plasma of the rat models of both insomnia and depression. A previous study showed that altered metabolites in animal models of sleep deprivation were primarily associated with amino acid (L-glutamic acid and tryptophan) metabolism (Bourdon et al., 2018). Furthermore, alterations in rhythmic metabolites have been observed in studies of patients with insomnia, with a reduction in branched-chain amino acid metabolites, mainly at night (Gehrman et al., 2018). Similarly, metabolic changes in models of depression are characterized by reduced neurotransmitters in the brain and amino acid metabolite levels in the blood (Pu et al., 2021). This is consistent with our study, in which we focused on the metabolic changes of GABA and L-Glutamic acid in the hippocampus of rats. We found that GABA levels were down-regulated in rats with insomnia and depression. L-Glutamic acid was enriched in the brains of insomnia rats, while a down-regulation trend was observed in depressed rats. Metabolite disorders can be partially ameliorated after SZRD treatment. The balance between glutamic acid and GABA is crucial in maintaining normal brain function (Duman et al., 2019). Research indicates that chronic stress may impair brain function in rats by diminishing the structural integrity and functionality of neurons that release glutamic acid and by lowering the metabolic activity of glutamic acid and GABA within the brain tissue (Banasr et al., 2010). Clinical studies have also reported reductions in glutamic acid and GABA markers in other psychiatric disorders, including schizophrenia and depression (Fee et al., 2017). Our study confirmed these findings with the down-regulation of Glu in the brain tissue of depression rats. The abnormal expression of glutamic acid and GABA in brain tissue may be influenced by several factors, including synthesis, metabolism, and reuptake by neurons and neuroglial cells (Lener et al., 2017). Overall, SZRD treatment partially restored metabolite levels in plasma and the hippocampus of rats with insomnia and depression.

**Figure 11:**
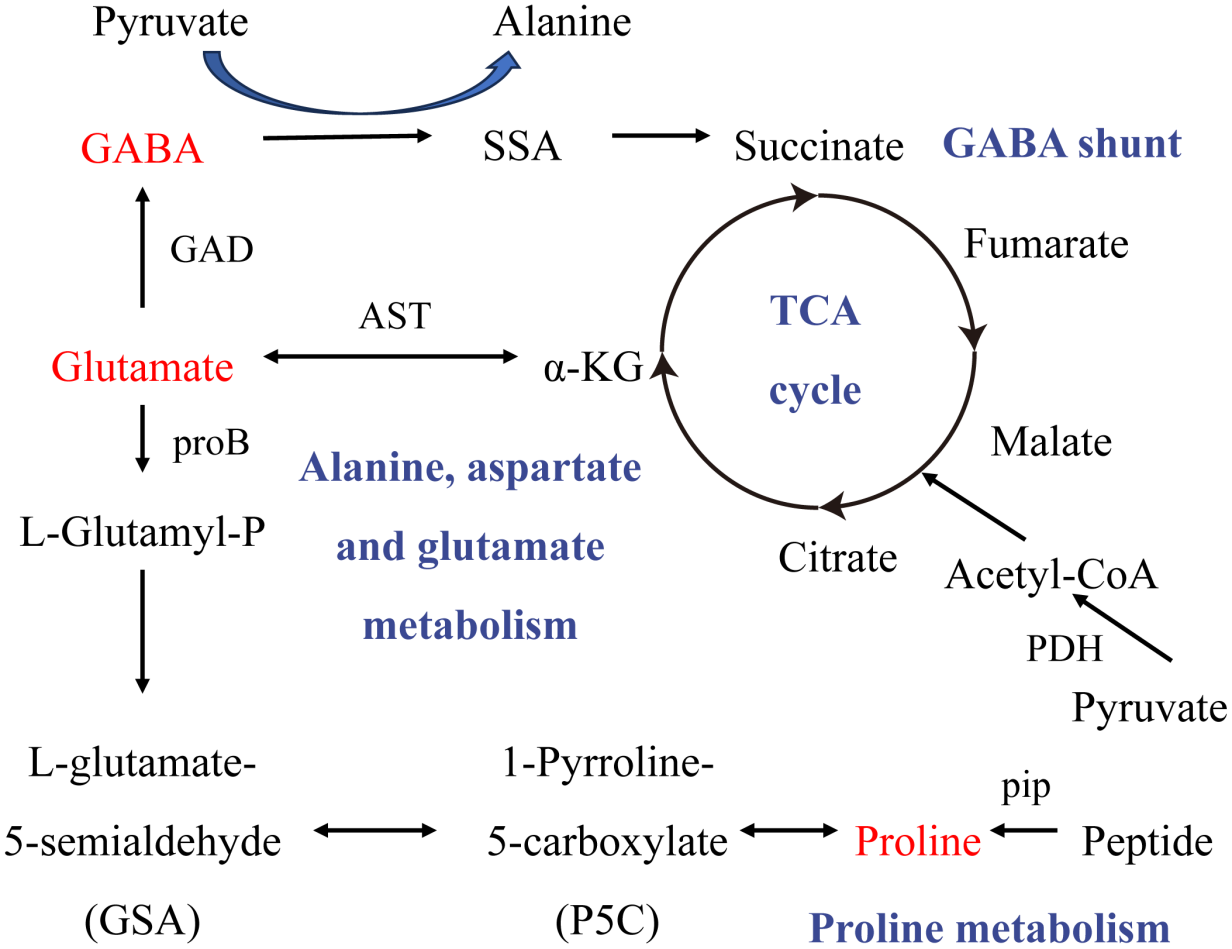
The alanine, aspartate, and glutamate metabolism pathway.

Correlation analysis showed that the brain metabolite L-Glutamic acid, the neurotransmitter GABA, and Lactobacillus play important roles in the process of insomnia. Similarly, in the development of depression, the blood metabolite L-Proline, the brain metabolite *γ*-aminobutyric acid, the neurotransmitter GABA, and Lactobacillus play crucial roles. KEGG pathway analysis revealed that the alanine, aspartate, and glutamate metabolism pathway was enriched in rats with insomnia and depression (Figure 11). Glutamic acid, L-Proline, and GABA were all involved in this pathway (Mayneris-Perxachs et al., 2022). These findings suggest that the alanine, aspartate, and glutamate metabolism pathway could be a significant target for future research on the mechanisms by which SZRD exerts its effects. Although we revealed differences in gut microbiome and metabolome between insomnia and depressed rats modulated by different doses of SZRD in that study, there is insufficient evidence to conclude that changes in gut microbiome and metabolites contribute to insomnia and depression. Further fecal microbiome transplantation (FMT) research is needed to reveal the direct link between the gut microbiome and insomnia and depression. In addition, in-depth studies on the alanine, aspartate, and glutamate metabolic pathways involved in glutamic acid and GABA are necessary to clarify their important roles in both insomnia and depression.

## 5 Conclusion

SZRD significantly improves the behavioral performance of rats with insomnia and depression. Specifically, low-dose SZRD (LSZRD) is more effective for insomnia, while high-dose SZRD (HSZRD) shows better results for depression. SZRD modulates gut microbiome homeostasis, increases the abundance of SCFA-producing genera, restores amino acid metabolite concentrations in plasma, and stabilizes γ-aminobutyric acid (GABA) and L-Glutamic acid levels in hippocampal tissues. These findings confirm that SZRD can intervene in different disease models of insomnia and depression through gut microbiome modulation and metabolomic pathways. This study provides a foundation for using LSZRD in treating insomnia and HSZRD in treating depression.

## Data Availability

The original contributions presented in the study are included in the article/Supplementary material, further inquiries can be directed to the corresponding authors.
