# Supplementary material for: Integrated gut microbiome and metabolomic analyses elucidate the therapeutic mechanisms of Suanzaoren decoction in insomnia and depression models
Source: Front Neurosci. 2024 Oct 11;18:1459141. doi: 10.3389/fnins.2024.1459141 (PMC11502468; doi:10.3389/fnins.2024.1459141)
Supplement: Supplementary file 1 [file Data_Sheet_1.PDF]

Supplementary Material

1 Supplementary Figures and Tables

1.1 Supplementary Tables

Table S1 66 components of SZRD by UHPLC-LTQ-Orbitrap-MS

No.	RT (min)	Compounds	Formula	Error (ppm)	m/z	MS^2 Fragmentation	Selected Ion	Compound types
1	5.11	Neomangiferin	$C_{25}H_{28}O_{16}$	-1.027	583.1288	565.2086、493.1188、463.1724、421.1395、403.1574、331.1326、301.0710	$[M-H]^-$	Flavonoid
2	6.98	5,7,3',4',5'-Pentahydroxyflavone	$C_{15}H_{10}O_7$	-0.331	301.0342	282.9821、272.9874、257.9858	$[M-H]^-$	Flavonoid
3	7.02	Coclaurine	$C_{17}H_{19}NO_3$	-2.453	286.1431	269.0526、237.1716、174.9793、106.8937	$[M+H]^+$	Alkaloid
4	7.16	Mangiferin	$C_{19}H_{18}O_{11}$	0.710	421.0768	403.0918、331.0645、301.0006	$[M-H]^-$	Flavonoid
5	7.51	Isomangiferin	$C_{19}H_{18}O_{11}$	0.237	421.0766	403.1936、331.0466、301.0348	$[M-H]^-$	Flavonoid
6	7.80	Magnoflorine	$C_{20}H_{23}NO_4$	-0.876	342.1697	297.0517、282.1034、279.0682、265.0783	$[M+H]^+$	Alkaloid
7	9.74	Isovitexin 2''-O-β-D-glucopyranoside	$C_{27}H_{30}O_{15}$	-2.019	595.1645	433.0998、415.2034、337.1197、313.1161、283.1795	$[M+H]^+$	Flavonoid
8	10.20	Isovitexin	$C_{21}H_{20}O_{10}$	-0.925	433.1125	397.3402、283.1970	$[M+H]^+$	Flavonoid
9	10.23	Vitexin	$C_{21}H_{20}O_{10}$	-1.850	433.1121	415.4339、397.3402、379.3412、283.1970	$[M+H]^+$	Flavonoid
10	10.30	Spinosin	$C_{28}H_{32}O_{15}$	-2.465	609.1799	447.1072、429.0953、411.1386、327.1232、297.0456	$[M+H]^+$	Flavonoid

11	10.32	Isospinosin	$C_{28}H_{32}O_{15}$	-2.465	609.1799	447.1732、327.0742、297.1045、285.0484	$[M+H]^+$	Flavonoid
12	10.37	Liquiritin	$C_{21}H_{22}O_9$	0.478	417.1182	255.0437、134.8830、119.0608	$[M-H]^-$	Flavonoid
13	10.38	4',7-Dihydroxyflavanone	$C_{15}H_{12}O_4$	-1.171	257.0805	242.0390、238.9686、210.9661、162.8893、146.8982、136.8845	$[M+H]^+$	Flavonoid
14	10.48	Liquiritin Apioside	$C_{26}H_{30}O_{13}$	-0.182	549.1602	417.1184、297.0256、255.0052	$[M-H]^-$	Flavonoid
15	10.51	Licuraside	$C_{26}H_{30}O_{13}$	-0.182	549.1602	417.1884、297.0256、255.0052	$[M-H]^-$	Flavonoid
16	10.69	Caaverine	$C_{17}H_{17}NO_2$	0.000	268.1332	251.0335、219.0218	$[M+H]^+$	Alkaloid
17	12.29	Kaempferol-3-O-rutinoside	$C_{27}H_{30}O_{15}$	-1.009	593.1495	285.0106、284.3292、255.0854	$[M-H]^-$	Flavonoid
18	13.76	Naringenin	$C_{15}H_{12}O_5$	-0.735	273.0756	152.9453、146.8999、118.9620	$[M+H]^+$	Flavonoid
19	13.80	5-Hydroxylliquiritin	$C_{21}H_{22}O_{10}$	0.230	433.1130	271.0694	$[M-H]^-$	Flavonoid
20	14.31	Senkyunolide I/H	$C_{12}H_{16}O_4$	-0.446	225.1120	207.0757	$[M+H]^+$	Phthalide
21	14.40	Senkyunolide F	$C_{12}H_{14}O_3$	-0.485	207.1015	189.0469、171.0117、160.9678	$[M+H]^+$	Phthalide
22	14.53	6′′′-Feruloylspinosin	$C_{38}H_{40}O_{18}$	-0.765	785.2281	351.1606、327.1074、297.0990	$[M+H]^+$	Flavonoid
23	14.75	6′′-Feruloylspinosin	$C_{38}H_{40}O_{18}$	-0.765	785.2281	447.0077、327.1555、285.2786	$[M+H]^+$	Flavonoid
24	15.31	3-Butenyl-4-hydroxybenzoate	$C_{12}H_{14}O_3$	-0.485	207.1015	188.9923、170.9175、132.8990	$[M+H]^+$	Phthalide
25	15.45	Timosaponin N	$C_{45}H_{76}O_{20}$	-1.067	981.4891	935.4365、773.6547	$[M+HCOO]^-$	Steroid
26	15.90	6‴-(N-β-D glucopyranosyl)-2″″, 3″″-dihydro-2″″-oxo-3″″ yl-acetate spinosin	$C_{44}H_{49}NO_{22}$	-0.318	944.2816	782.2027、764.2195、602.1777、488.9156、393.1713、351.2115、327.1060	$[M+H]^+$	Flavonoid
27	16.04	Isoliquiritin Apioside	$C_{26}H_{30}O_{13}$	-0.545	549.1600	417.1489、297.0561、255.0820	$[M-H]^-$	Flavonoid
28	16.09	epi-6'''-(N-β-D glucopyranosyl)-2'''',3''''- dihydro-2''''-oxo-3''''-yl acetate spinosin	$C_{44}H_{49}NO_{22}$	0.742	944.2826	782.1841、764.1762、602.2708、489.0305、393.1843、375.1724、351.2294、327.1693	$[M+H]^+$	Flavonoid

29	16.35	Formononetin glucoside	$C_{22}H_{22}O_9$	0.000	475.1235	267.0067、252.0948	$[M+HCOO]^-$	Flavonoid
30	16.45	Isoliquiritoside	$C_{21}H_{22}O_9$	0.000	417.1180	255.0727、134.8676、119.0544	$[M-H]^-$	Flavonoid
31	16.65	2,6,4'-Trihydroxy-4-methoxybenzophenone	$C_{14}H_{12}O_5$	0.769	259.0603	164.9092、149.8589	$[M-H]^-$	Phenol
32	16.81	Nornuciferine	$C_{18}H_{19}NO_2$	-0.711	282.1487	265.0693	$[M+H]^+$	Alkaloid
33	16.94	Liquiritigenin	$C_{15}H_{12}O_4$	1.171	255.0655	134.8955、118.8446、90.7955	$[M-H]^-$	Flavonoid
34	17.37	Neoliquiritin	$C_{21}H_{22}O_9$	0.000	417.1180	255.0748、134.8478、118.9935	$[M-H]^-$	Flavonoid
35	18.26	Licorice glycoside B	$C_{35}H_{36}O_{15}$	-2.010	695.1956	549.1909、531.2285、255.0638	$[M-H]^-$	Flavonoid
36	18.69	Barpisoflavone A	$C_{16}H_{12}O_6$	0.000	299.0550	284.0101、271.1573、255.0786	$[M-H]^-$	Flavonoid
37	19.92	Timosaponin Bii	$C_{45}H_{76}O_{19}$	-1.086	965.4942	919.5649、757.6413	$[M+HCOO]^-$	Steroid
38	20.40	petunioside N	$C_{51}H_{86}O_{24}$	-3.512	1081.5387	919.4700、757.4731	$[M-H]^-$	Steroid
39	22.90	Amphibin D	$C_{36}H_{49}N_5O_5$	-2.532	632.3790	344.2807、289.0506	$[M+H]^+$	Alkaloid
40	23.59	Licoricesaponin A3	$C_{48}H_{72}O_{21}$	-2.639	983.4456	821.4553、351.0230	$[M-H]^-$	Triterpene
41	24.10	3-Butylidenephthalide	$C_{12}H_{12}O_2$	-1.063	189.0908	170.9592、160.9350、142.9537	$[M+H]^+$	Phthalide
42	24.20	Timosaponin Biii	$C_{45}H_{74}O_{18}$	-0.443	947.4842	901.5334	$[M+HCOO]^-$	Steroid
43	24.64	Isoliquiritigenin	$C_{15}H_{12}O_4$	0.780	255.0654	134.8680、118.9094、90.8457	$[M-H]^-$	Flavonoid
44	24.86	Formononetin	$C_{16}H_{12}O_4$	0.000	269.0808	254.0056、237.0028、213.0698、106.8313	$[M+H]^+$	Flavonoid
45	25.26	Dehydrotrametenolic acid	$C_{30}H_{46}O_3$	-2.639	455.3508	437.3734、295.2408	$[M+H]^+$	Triterpene
46	25.66	Licoricesaponin G2	$C_{42}H_{62}O_{17}$	0.238	839.4062	663.4094、487.4085、469.3397	$[M+H]^+$	Triterpene
47	26.25	Anemarrhenasaponin I	$C_{39}H_{66}O_{14}$	-1.054	803.4416	595.4402、433.5726	$[M+HCOO]^-$	Steroid
48	26.36	Jujuboside B	$C_{52}H_{84}O_{21}$	-2.296	1089.5452	1043.5824	$[M+HCOO]^-$	Triterpene
49	26.43	Glycyrrhizic acid	$C_{42}H_{62}O_{16}$	-0.972	821.3946	803.4496,645.3517,351.1814	$[M-H]^-$	Triterpene

50	26.68	Senkyunolide B	$C_{12}H_{12}O_3$	2.448	203.0708	173.8690、159.8237、144.8206	$[M-H]^-$	Phthalide
51	26.82	Ferulic acid	$C_{10}H_{10}O_4$	3.090	193.0501	192.9565、177.9515、148.9057	$[M-H]^-$	Organic acids
52	27.21	Timosaponin G	$C_{39}H_{64}O_{14}$	-0.528	801.4263	755.3830、593.2020	$[M+HCOO]^-$	Steroid
53	27.74	Senkyunolide A	$C_{12}H_{16}O_2$	-3.121	193.1217	175.0717、165.0605、146.9265、136.8648、122.9128、119.0066、104.9360	$[M+H]^+$	Phthalide
54	28.31	Glycycoumarin	$C_{21}H_{20}O_6$	-0.271	367.1175	352.0494、309.0132	$[M-H]^-$	Flavonoid
55	29.28	Licoisoflavanone	$C_{20}H_{18}O_6$	-0.282	353.1019	309.2115、285.1258、227.0393、199.0198、124.8594	$[M-H]^-$	Flavonoid
56	29.40	Poricoic acid A	$C_{31}H_{46}O_5$	-0.802	497.3258	479.2956、423.4944	$[M-H]^-$	Triterpene
57	29.76	Glycyrol	$C_{21}H_{18}O_6$	-2.729	365.1010	307.0854、295.0621	$[M-H]^-$	Flavonoid
58	29.85	Butylphthalide	$C_{12}H_{14}O_2$	-2.628	191.1062	172.8945、144.9682、134.9332、116.8573	$[M+H]^+$	Phthalide
59	29.89	Ligustilide	$C_{12}H_{14}O_2$	-2.628	191.1062	172.9666、162.9991、148.9679、144.9362	$[M+H]^+$	Phthalide
60	30.12	Licoflavone C	$C_{20}H_{18}O_5$	-0.887	337.1068	293.1371、281.2095、253.0231	$[M-H]^-$	Flavonoid
61	30.38	Timosaponin A-III	$C_{39}H_{64}O_{13}$	-0.675	785.4313	739.4454、577.2430	$[M+HCOO]^-$	Steroid
62	31.01	Semilicoisoflavone B	$C_{20}H_{16}O_6$	-1.419	353.1015	335.0583、325.0342、311.0695、299.1078	$[M+H]^+$	Flavonoid
63	31.25	26-hydroxyporicoic acid G	$C_{30}H_{46}O_6$	-0.797	501.3207	471.3183、457.1807、439.5161	$[M-H]^-$	Triterpene
64	32.80	3β,16α-dihydroxylanosta 7,9(11),24-trien-21-oic acid	$C_{30}H_{46}O_4$	-0.213	469.3311	449.0983、451.3840、407.3343	$[M-H]^-$	Triterpene
65	33.02	Ceanothic acid	$C_{30}H_{46}O_5$	0.205	485.3263	439.4104、423.3945	$[M-H]^-$	Triterpene
66	33.81	Trametenolic acid	$C_{30}H_{48}O_3$	0.000	455.3520	437.5790、435.2104、339.0368	$[M-H]^-$	Triterpene

Tabel S2 Correlation network analysis of Suanzaoren decoction in intervening insomnia model rats

ID	Category	Degree	Weighted Degree	Betweenness Centrality
L-Glutamic acid	Brain metabolites	16	11.21897526	82.55
2-Hydroxyisobutyric acid	Brain metabolites	9	6.130431487	44.88333333
L-Serine	Blood metabolites	8	5.372503219	7.083333333
3-Hydroxybutyric acid	Brain metabolites	8	5.471062329	9.166666667
L-Iditol	Brain metabolites	8	5.679796857	8.583333333
Lactobacillus	Microbiota	7	4.583527537	22.58333333
Malonic acid	Brain metabolites	7	4.85956438	1.916666667
GABA	Neurotransmitter	6	3.926993438	7.3
Glu	Neurotransmitter	6	4.073605424	21.91666667
Oleic Acid	Brain metabolites	6	4.218379815	1.25
Ascorbic acid	Brain metabolites	6	4.306414373	0.833333333
Prevotella	Microbiota	5	3.262156877	2.333333333
L-Threonine	Blood metabolites	5	3.515469605	1.466666667
γ-aminobutyric acid	Brain metabolites	5	3.159002824	0.666666667
Bacteroides	Microbiota	4	2.494066306	2.466666667
Aconitic acid	Blood metabolites	4	2.751218376	20
L-Valine	Blood metabolites	2	1.330988464	20
DA	Neurotransmitter	2	1.244134282	0
Akkermansia	Microbiota	1	0.62333853	0
Clostridium_IV	Microbiota	1	0.688216417	0
Peptococcus	Microbiota	1	0.622385363	0
NE	Neurotransmitter	1	0.814821886	0
Urea	Blood metabolites	1	0.958577713	0
Stearic acid	Blood metabolites	1	0.715542522	0
Ruminococcus	Microbiota	1	0.62333853	0
Phascolarctobacterium	Microbiota	1	0.602016509	0
Oxalic acid	Blood metabolites	1	0.958577713	0
D-Mannose	Brain metabolites	1	0.715542522	0

Tabel S3 Correlation network analysis of Suanzaoren decoction in intervening depression model rats

ID	Category	Degree	Weighted Degree	Betweenness Centrality
L-Proline	Blood metabolites	26	35.52187616	196.1933583
γ-aminobutyric acid	Brain metabolites	24	33.20941431	75.33785426
Lactic acid	Blood metabolites	21	28.99415573	58.33517187
L-Threonine	Blood metabolites	20	28.26884214	35.24870985
Glycerol	Brain metabolites	16	20.94421725	57.33541966
L-Valine	Brain metabolites	16	22.02129707	16.70866446

Lactobacillus	Microbiota	15	20.36094103	21.43060244
L-Isoleucine	Blood metabolites	15	20.82184751	14.58143584
Blood L-Serine	Blood metabolites	15	20.35997067	17.05145426
GABA	Neurotransmitter	14	18.93981199	40.67813962
Brain L-Alanine	Brain metabolites	14	18.54484532	18.03618798
Brain L-Aspartic acid	Brain metabolites	14	19.9592905	7.52375779
9-Hexadecenoic acid	Blood metabolites	12	15.98001971	26.96458874
Citric acid	Blood metabolites	11	15.1297654	54.65957963
DA	Neurotransmitter	11	14.62125569	17.93060273
Glycine	Brain metabolites	11	16.01172448	3.440414249
Creatinine	Brain metabolites	11	14.82771261	3.446772569
L-Leucine	Blood metabolites	10	13.72687411	4.722525063
NE	Neurotransmitter	9	12.00220047	1.121500722
Helicobacter	Microbiota	7	8.606426475	24.13380231
Blood L-Aspartic acid	Blood metabolites	7	9.284568216	10.93372294
Prevotella	Microbiota	7	8.80058651	47.14378816
Roseburia	Microbiota	7	8.857881731	18.62988539
Cholesterol	Brain metabolites	7	9.752199413	0.549197236
L-Glutamic acid	Brain metabolites	7	8.870967742	0.111111111
Clostridium_IV	Microbiota	6	7.613385203	19.09193723
Barnesiella	Microbiota	6	7.816576103	2.776017155
Brain L-Serine	Brain metabolites	6	7.595307918	0.188034188
Intestinimonas	Microbiota	5	6.359721127	0.938034188
Paraprevotella	Microbiota	5	6.213343109	14.50800866
Glu	Neurotransmitter	5	6.367519044	1.568572071
Clostridium_XlVa	Microbiota	4	4.895894428	42.91944444
Myo-Inositol	Blood metabolites	4	5.024926686	6.937637363
Blood L-Alanine	Blood metabolites	3	3.752932551	4.490734163
Alistipes	Microbiota	2	2.490469208	0.333333333
Oscillibacter	Microbiota	2	2.673020528	0
5-HT	Neurotransmitter	2	2.670234777	0
3-Hydroxybutyric acid	Blood metabolites	2	2.889296188	0
Alloprevotella	Microbiota	1	1.367302053	0
Bacteroides	Microbiota	1	1.214809384	0
Lachnospiracea_incertae_sedis	Microbiota	1	1.269061584	0

1.2 Supplementary Figures

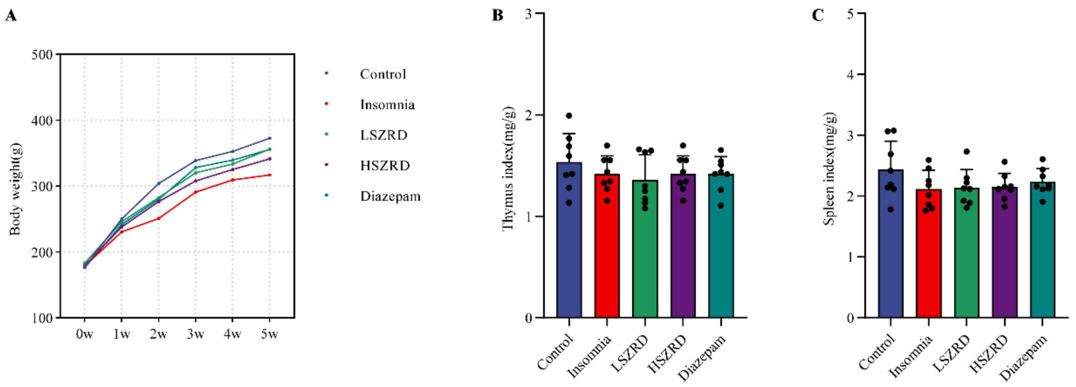

Figure. S1 The body weight and organ index of insomnia rats (n =8). **A** Body weight. **B-C** The thymus and spleen indexes.

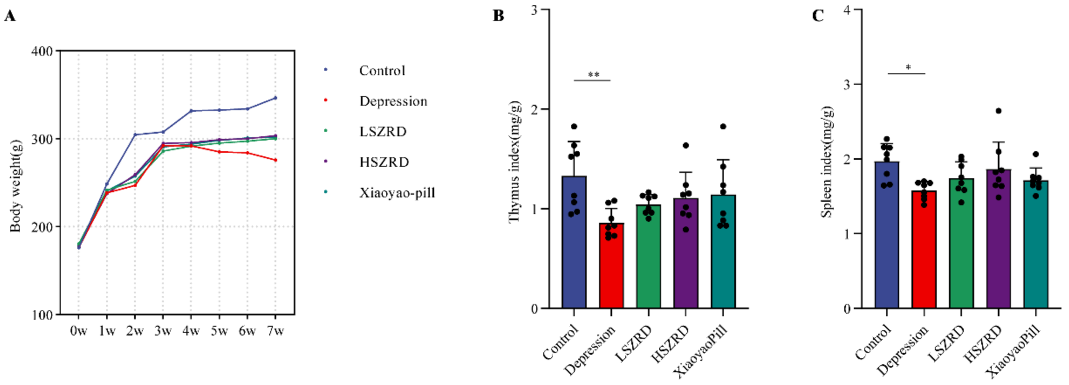

Figure. S2 The body weight and organ index of depression rats (n =8). **A** Body weight. **B-C** The thymus and spleen indexes. $^{*}P < 0.05$, $^{**}P < 0.01$.

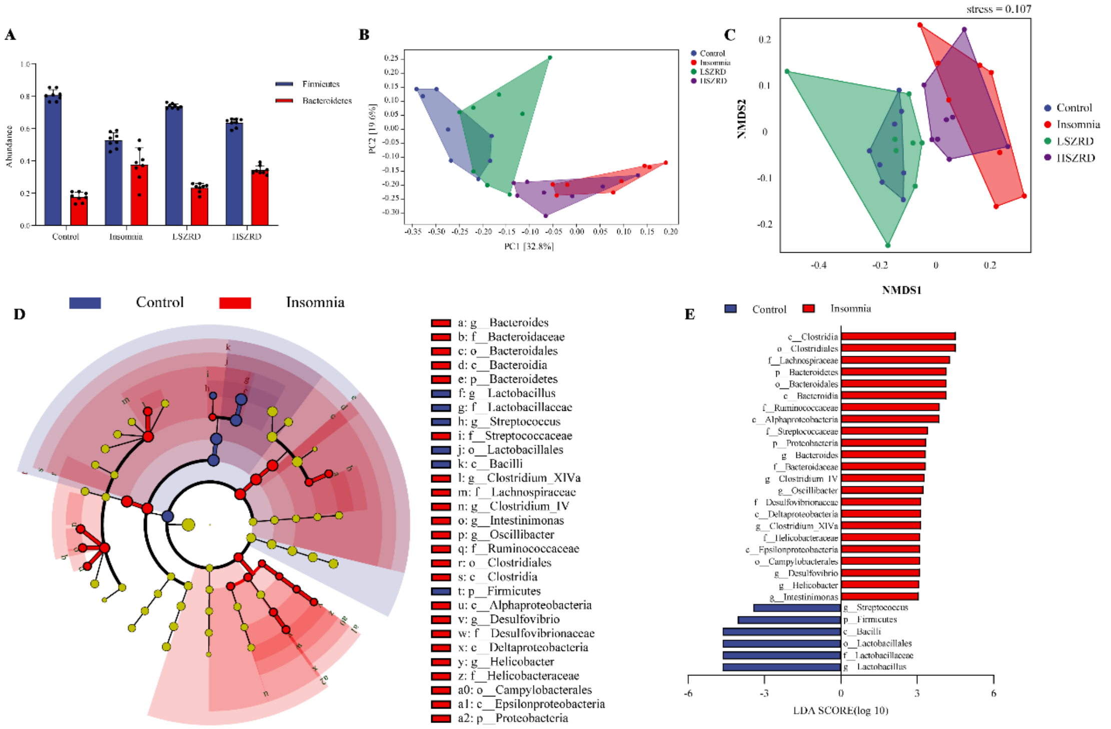

Figure. S3 SZRD partially ameliorated gut microbiome disorders in insomnia rats (n =8). **A** Abundance of the Firmicutes and Bacteroidetes in each group. **B** PCoA plot. **C** NMDS plot. **D** The LEfSe analysis of Control and Insomnia groups. **E** LDA histogram of the Control and Insomnia groups.

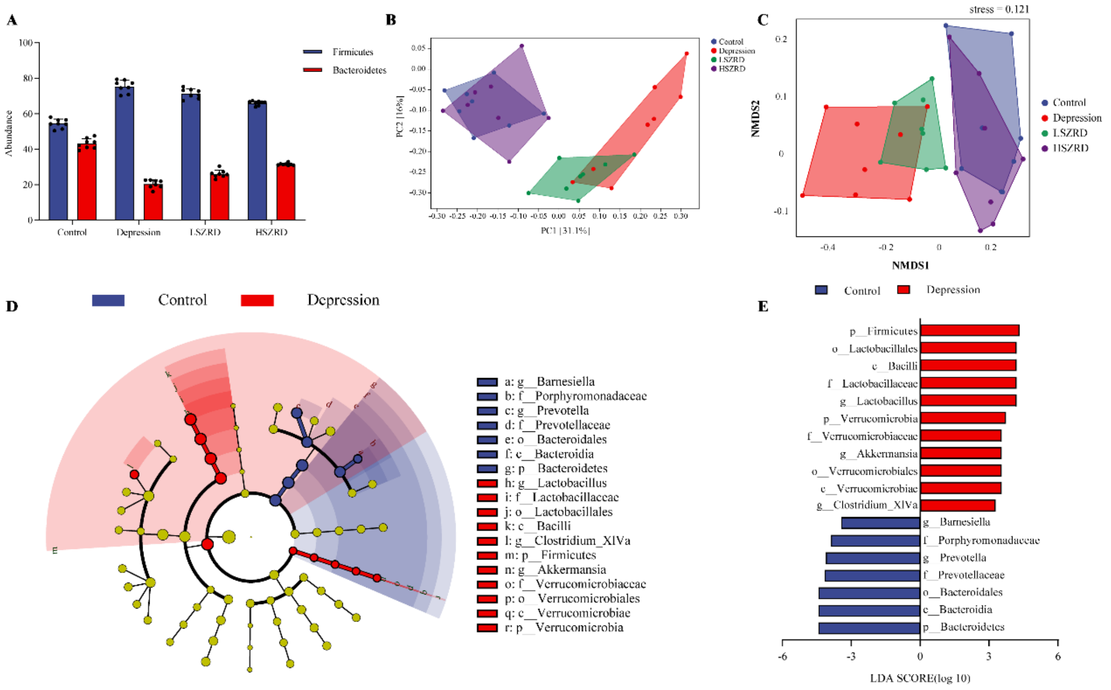

Figure. S4 SZRD partially ameliorated gut microbiome disorders in depression rats (n =8). **A** Abundance of the Firmicutes and Bacteroidetes in each group. **B** PCoA plot. **C** NMDS plot. **D** The LEfSe analysis of Control and Depression groups. **E** LDA histogram of the Control and Depression groups.

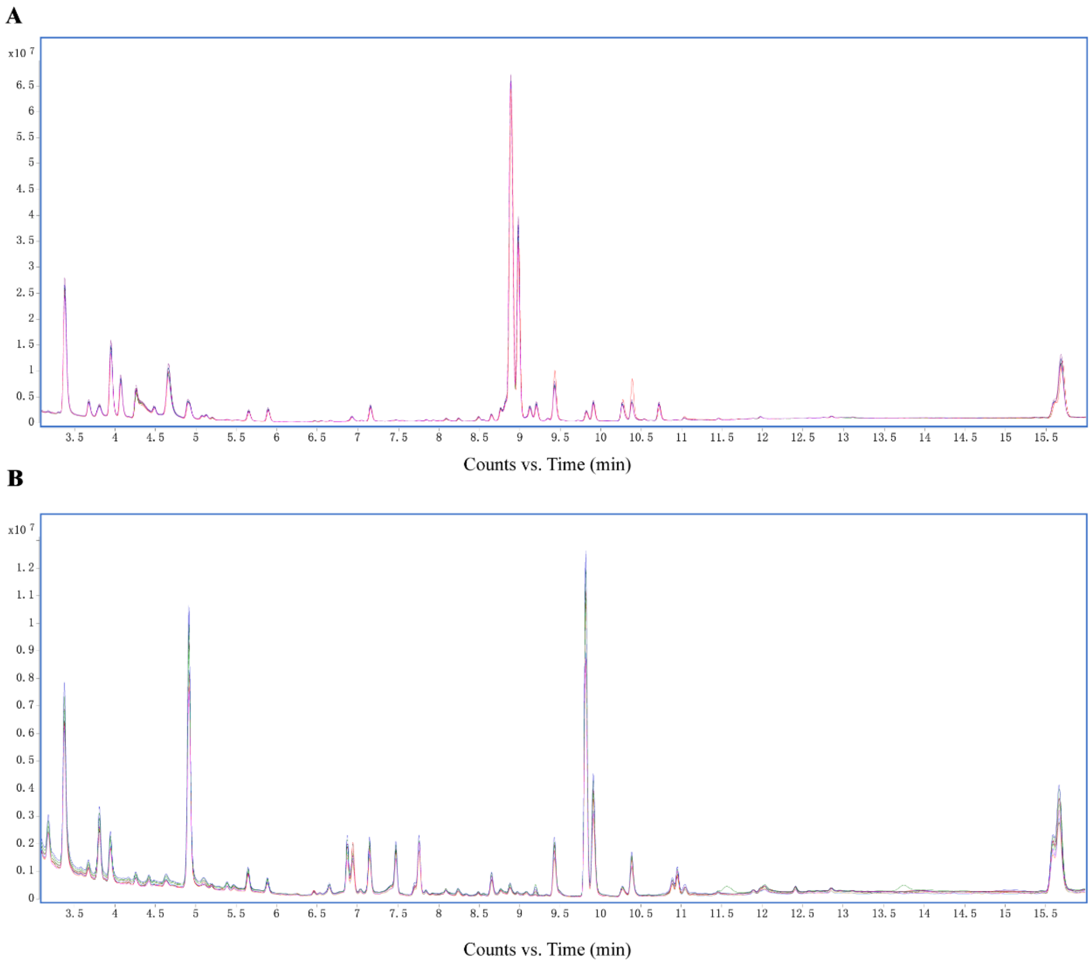

Figure. S5 The chromatogram of QC samples. **A** Plasma metabolomics. **B** Hippocampus metabolomics.

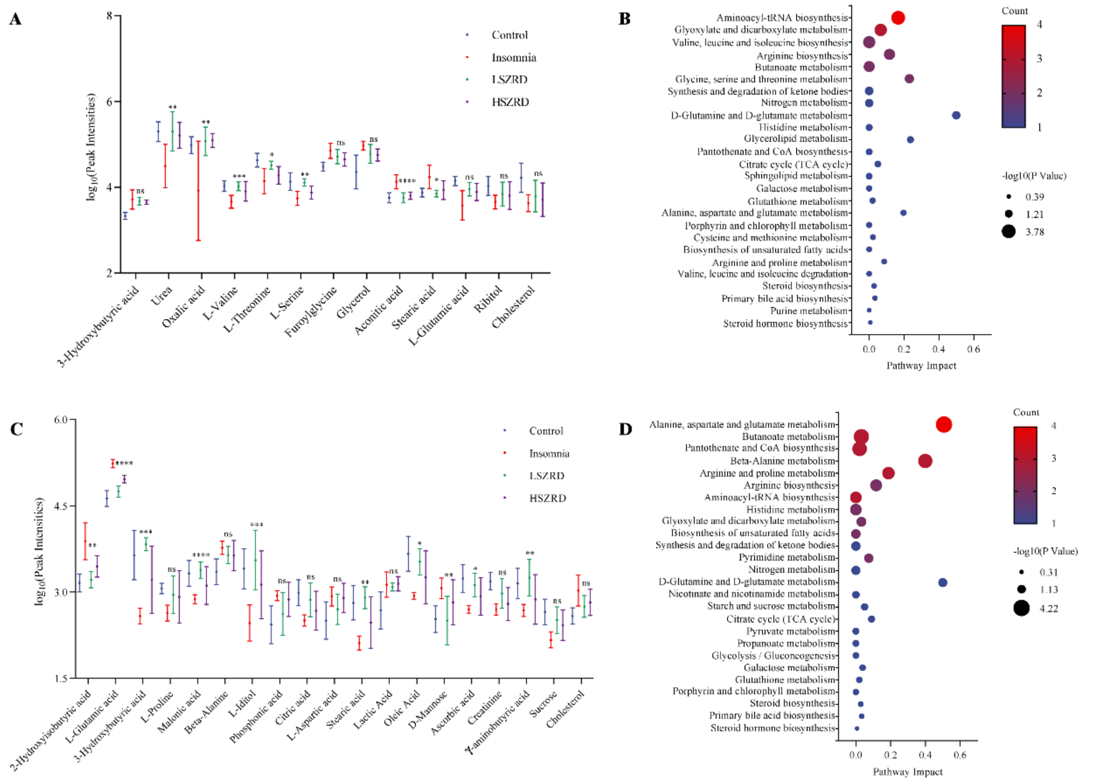

Figure. S6 Impacts of LSZRD on metabolites in insomnia rats. Plasma metabolomics analysis: **A** Differential analysis of metabolites among the four groups. **B** KEGG pathway enrichment analysis bubble plot. Hippocampus metabolomics analysis: **C** Differential analysis of metabolites. **D** KEGG pathway enrichment analysis. $^{*}P < 0.05$, $^{**}P < 0.01$, $^{***}P < 0.001$, $^{****}P < 0.0001$; ns, no significant difference; n = 8.

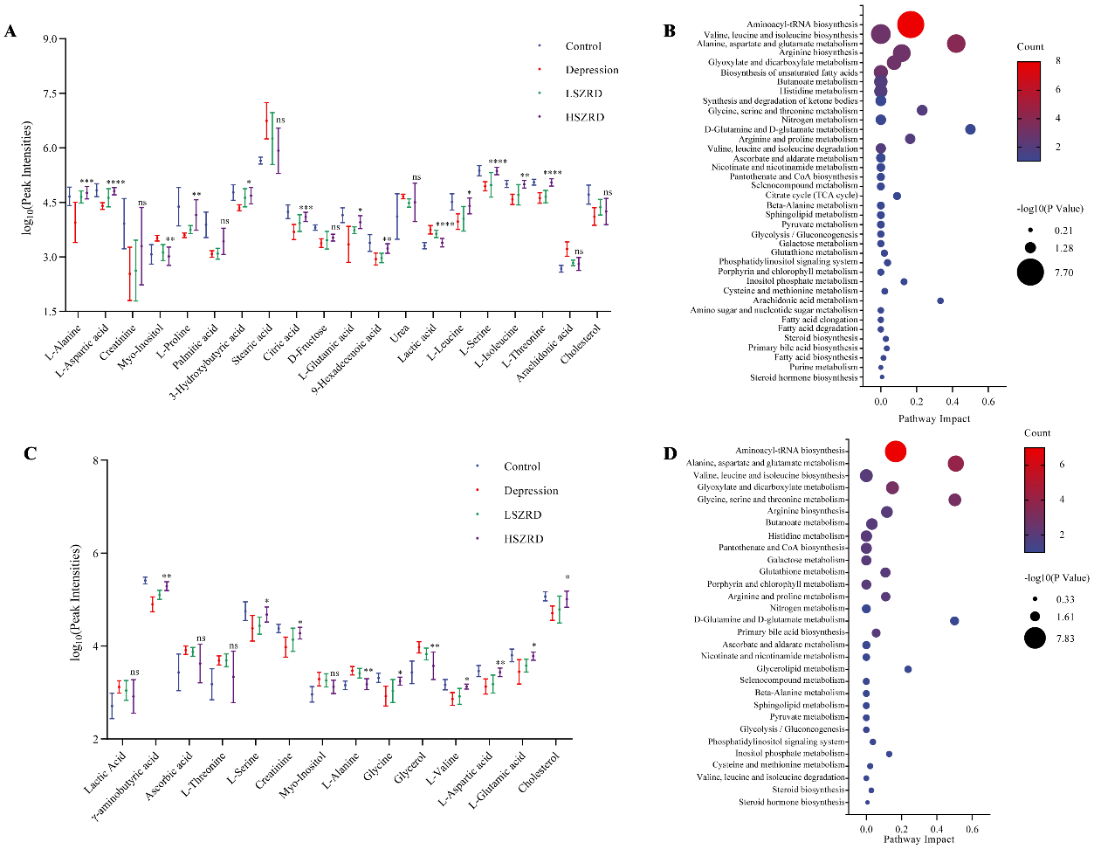

Figure. S7 Impacts of HSZRD on metabolites in the rats caused by CUMS. Plasma metabolomics analysis: **A** Differential analysis of metabolites among the four groups. **B** KEGG pathway enrichment analysis bubble plot. Hippocampus metabolomics analysis: **C** Differential analysis of metabolites. **D** KEGG pathway enrichment analysis. $^{*}P < 0.05$, $^{**}P < 0.01$, $^{***}P < 0.001$, $^{****}P < 0.0001$; ns, no significant difference; n = 8.
